# Variation and convergence in the morpho-functional properties of the mammalian neocortex

**DOI:** 10.3389/fnsys.2024.1413780

**Published:** 2024-06-20

**Authors:** Séverine Mahon

**Affiliations:** Sorbonne Université, Institut du Cerveau - Paris Brain Institute - ICM, Inserm, CNRS, APHP, Hôpital de la Pitié Salpêtrière, Paris, France

**Keywords:** neocortex, pyramidal neurons, cortical circuits, intrinsic excitability, brain rhythms, mammals, evolution

## Abstract

Man's natural inclination to classify and hierarchize the living world has prompted neurophysiologists to explore possible differences in brain organisation between mammals, with the aim of understanding the diversity of their behavioural repertoires. But what really distinguishes the human brain from that of a platypus, an opossum or a rodent? In this review, we compare the structural and electrical properties of neocortical neurons in the main mammalian radiations and examine their impact on the functioning of the networks they form. We discuss variations in overall brain size, number of neurons, length of their dendritic trees and density of spines, acknowledging their increase in humans as in most large-brained species. Our comparative analysis also highlights a remarkable consistency, particularly pronounced in marsupial and placental mammals, in the cell typology, intrinsic and synaptic electrical properties of pyramidal neuron subtypes, and in their organisation into functional circuits. These shared cellular and network characteristics contribute to the emergence of strikingly similar large-scale physiological and pathological brain dynamics across a wide range of species. These findings support the existence of a core set of neural principles and processes conserved throughout mammalian evolution, from which a number of species-specific adaptations appear, likely allowing distinct functional needs to be met in a variety of environmental contexts.

## 1 Introduction

Questions about the specific properties of the human brain originated in the debates that followed the publication of Darwin (1859)'s *Origin of Species*. Challenging long-held beliefs, largely inherited from the biblical Genesis account, that humans were by essence different from other animals, the application of the theory of evolution to humans soon gave rise to lively discussions about the biological traits likely to differentiate humans from apes and stimulated the first thoughts about the relationship between brain size and cognitive ability. Considered a good indicator of intelligence by most scientists in the second half of the 19^th^ century, overall brain size eventually proved to be an irrelevant measure of behavioural complexity, prompting the exploration of other levels of brain organisation. Thanks to *in vitro* investigations on postoperative tissue, our knowledge of the human neocortex—the cerebral structure whose functioning is critically involved in behavioural and cognitive abilities—has progressed considerably in recent years, providing the basis for inter-species comparisons at multiple levels. After tracing the historical origins of these questions back to the controversies surrounding the first discoveries of human fossils, this review is intended to provide an updated view of the functional organisation of the neocortex in representatives of the three major mammalian radiations, including humans. We will first analyze variations in brain size, number of cortical areas and number of neurons at a macroscopic level, before focusing on the anatomical and physiological features of neocortical pyramidal neurons. Adopting a reductionist approach, we will systematically compare (when data permit) the morphology, connectivity and electrical properties of pyramidal neuron subtypes between species, attempting to determine, where differences are observed, whether they are part of a continuum of variations or whether they represent genuine singularities leading to significant changes in the activity patterns of cortical circuits.

## 2 Eugene Dubois and the missing link

Proponents of Darwin's theory, led by the German biologist Ernst Haeckel, published essays in the 1860s discussing the status of the human species from an evolutionary point of view. According to Haeckel, the process of hominization was based on the acquisition of bipedalism, language and a large brain. In his family tree of the human species, which he attempted to reconstruct on the basis of comparative anatomical and embryological data, Haeckel inserted an intermediate evolutionary stage between the great apes and man, occupied by a hypothetical species called *Pithecanthropus* (ape-man) *alalus* (speechless). He imagined that this mute ape-man, originating from a continent now sunk in the Indian Ocean (*Lemuria*), could have spread and evolved in different parts of the world to give rise to different humanities and languages (Haeckel, 1868). Inspired by the work of Darwin and Haeckel and convinced of the necessity to support the theory of evolution with palaeontological evidence, in 1887 the young anatomist Eugène Dubois took the surprising decision to quit a promising academic career at the University of Amsterdam to mount an excavation campaign in the Dutch East Indies in search of the missing link between apes and humans. Accompanied by his wife Anna Lojenga and their daughter, Dubois enlisted as a medical officer in the Royal East India Army and set sail for the Indonesian archipelago aboard the *Princess Amelia* (Theunissen, 1988; Wood, 2020).

**Figure 1:**
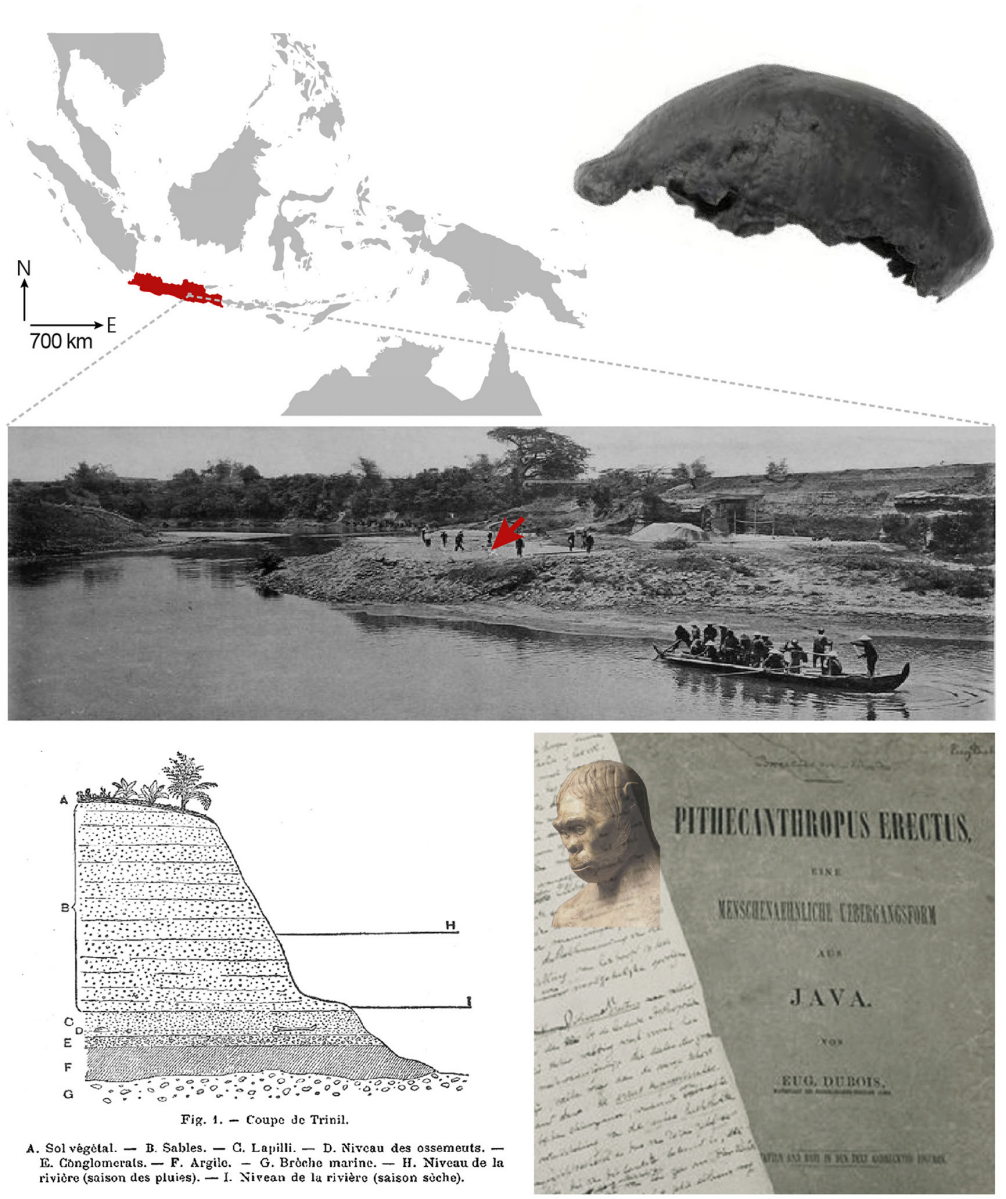
**Top left**, The Island of Java in the Indonesian archipelago. **Right**, The skull cap of *Pithecanthropus erectus*. **Middle**, Excavations at Trinil in 1890s on the left bank of the Solo River. The arrow indicates the approximate discovery site of the skull cap. **Bottom left**, Profile drawing of the Trinil site showing the position of the fossil remains in the sediment (level D). **Right**, Handwritten page from Dubois' article published in 1894 and photograph (John Reader/Science Photo Library) of his reconstruction of *Pithecanthropus erectus*, shown at the 1900 Universal Exhibition in Paris. Adapted with permission from Wood, 2020, and Dubois, 1896.

After arriving on the island of Java, Dubois conducted extensive excavations in the summer of 1891 near the village of Trinil, along the Solo River (Figure 1). He initially uncovered a right maxillary third molar and a skull cap whose characteristics—a receding forehead, a supra-orbital torus and an estimated capacity of 700–750 cm^3^ (about half the size of present-day humans)—suggested a large ape. However, the next year, Dubois unearthed a left femur some 15 metres upstream from the first remains, showing clear adaptations to upright posture and bipedalism (Theunissen, 1988). By discovering an individual whose skull and teeth displayed anthropoid ape characteristics but whose femur showed human-like features, Dubois had just uncovered fossil evidence of the hypothetical transitional primate envisioned by Haeckel, and at the same time provided one of the first material indications of human evolution. Initially named *Anthropithecus erectus* (an ape that stands and moves like a man) in the excavation reports, Dubois later renamed his new species *Pithecanthropus erectus*, emphasising its status as an upright “ape-man,” when he published his final manuscript (Figure 1). In good faith, he even revised the cranial capacity of his specimen to 850–900 cm^3^ (Dubois, 1894, 1896; Wood, 2020).

The discovery of *Pithecanthropus erectus* sparked controversy, particularly over the attribution of the remains to an ape, a human, an intermediate being, or even to different individuals. Dubois had to face the scepticism of his prominent European colleagues who would have preferred an ancestor with a larger brain but less exotic origins (Leguebe, 1992), such as the Piltdown Man discovered in Sussex in 1912, which had a large human-like skull and an ape-like mandible, but which ultimately turned out to be one of the greatest paleoanthropological hoaxes (Stringer, 2012). In response to debates over the interpretation of his fossils, Dubois undertook research on the allometric relationship between brain weight and body weight. Extending earlier theoretical works (Snell, 1892), he established that brain size was not only related to body weight by a decreasing power function but also depended on a “coefficient of cephalization,” supposed to reflect the degree of development and complexification of the brain (Dubois, 1897). Applying this mathematical relationship to his fossils, Dubois calculated that the cephalization coefficient of *Pithecantropus erectus* was roughly half that of anatomically modern humans and double that of apes, further confirming the intermediate evolutionary position of the Javanese primate (Dubois, 1899).

*Pithecanthropus erectus* is no longer considered the missing link. The idea of a hybrid creature, half ape and half human, making a direct transition between great apes and modern man, now belongs to the realm of fiction. The accumulation of fossil discoveries has led researchers to abandon the traditional vision of a linear and directed human evolution in favour of a more complex and diversified human lineage, made up of multiple species that most often coexisted. The concept of the missing link, which encompasses both the notion of continuity and rupture, remains pertinent as it questions the singularity of the “after” in relation to the “before.” Man is often seen as a species apart, distinguished by the complexity of its cultures, social interactions and ability to communicate. A species whose considerable brain growth over time would have accompanied the emergence of remarkable cognitive capacities, enabling man to conquer and transform almost all of the terrestrial ecosystems. But the question remains: does our large brain possess truly distinctive properties? And if so, are these differences merely quantitative or do they represent a genuine qualitative leap?

## 3 The long-standing question of size

Questions of absolute or relative brain size seem to have preoccupied mankind long before Dubois and his contemporaries since they already appear in the writings of Aristotle, who notes that “of all animals, man has the largest brain in proportion to his size” (Aristotle, ca. 335 BCE); a statement that is not entirely accurate, as we shall see below. However, it was mainly in the second half of the 19^th^ century that the relationship between brain size and human cognitive ability became a central theme of discussion among the scientific community, particularly within the *Société d'Anthropologie de Paris*, founded by the eminent neuroanatomist Paul Broca the year Darwin published his *Origin of Species*. A partisan of polygenist theories, which, in opposition to the creationist myth, favoured a multiple origin for the different human groups, but unfortunately mired in the prejudices of his time, Broca mistakenly thought that he could rank ethnic groups (and human beings in general) according to their level of intelligence by comparing the weight of their brains. By sorting individuals according to their ethnic origin, sex or profession, Broca came to the conclusion that white European men, whom he described as “distinguished” (as opposed to manual workers), were endowed with superior intelligence (Broca, 1861). In a similar vein, Francis Galton, an anthropologist renowned for his contributions to modern statistics and, more infamously, for his eugenics theories, later conducted a study into the brain size of Cambridge students. He also claimed, on the basis of measurements of questionable rigour, that those who graduated with honours had larger brains than those who did not receive such distinction (Galton, 1889).

In the wake of phrenology, which claimed to determine character traits and mental faculties by inspecting the size of bumps on the surface of skulls, the underlying—perhaps somewhat simplistic—assumption behind these early attempts to explain differences in cognitive ability by brain size was that any increase in the size of an organ should correspond to an increase in its function. Craniometric studies based on the social or geographical origins of human beings, which served as scientific justification for the expansionist ambitions of many countries in the 19^th^ and 20^th^ centuries, fortunately declined after the Second World War. However, the search for principles behind the evolution of the mammalian brain has never ceased to intrigue scientists, who continue to study variations in absolute and relative brain size between species, as well as the evolution of its different parts, paying particular attention to the neocortex because of its essential role in the generation of complex behaviours.

### 3.1 Brain size in mammals

As neuronal tissue does not fossilise, the formulation of general principles on the evolution of the brain requires a comparative analysis of cerebral organisation in living species (and, to a lesser extent, the study of endocranial casts of fossil specimens), based on the hypothesis that the characteristics present in the current members of a phylogenetic radiation can be explained more parsimoniously as being inherited from a common ancestor. The earliest mammals have likely evolved from mammal-like reptiles at the end of the Triassic over 200 million years ago, in the form of small-brained shrew-like creatures that probably laid eggs, like present-day monotremes (Kaas, 2011). Monotremes (prototherians), one of the three main extant mammalian groups, diverged from therians around 166–186 million years ago, while the marsupial (metatherian) and placental (eutherian) lineages are thought to have split around 147–160 million years ago (Bininda-Emonds et al., 2007; Phillips et al., 2009).

**Figure 2:**
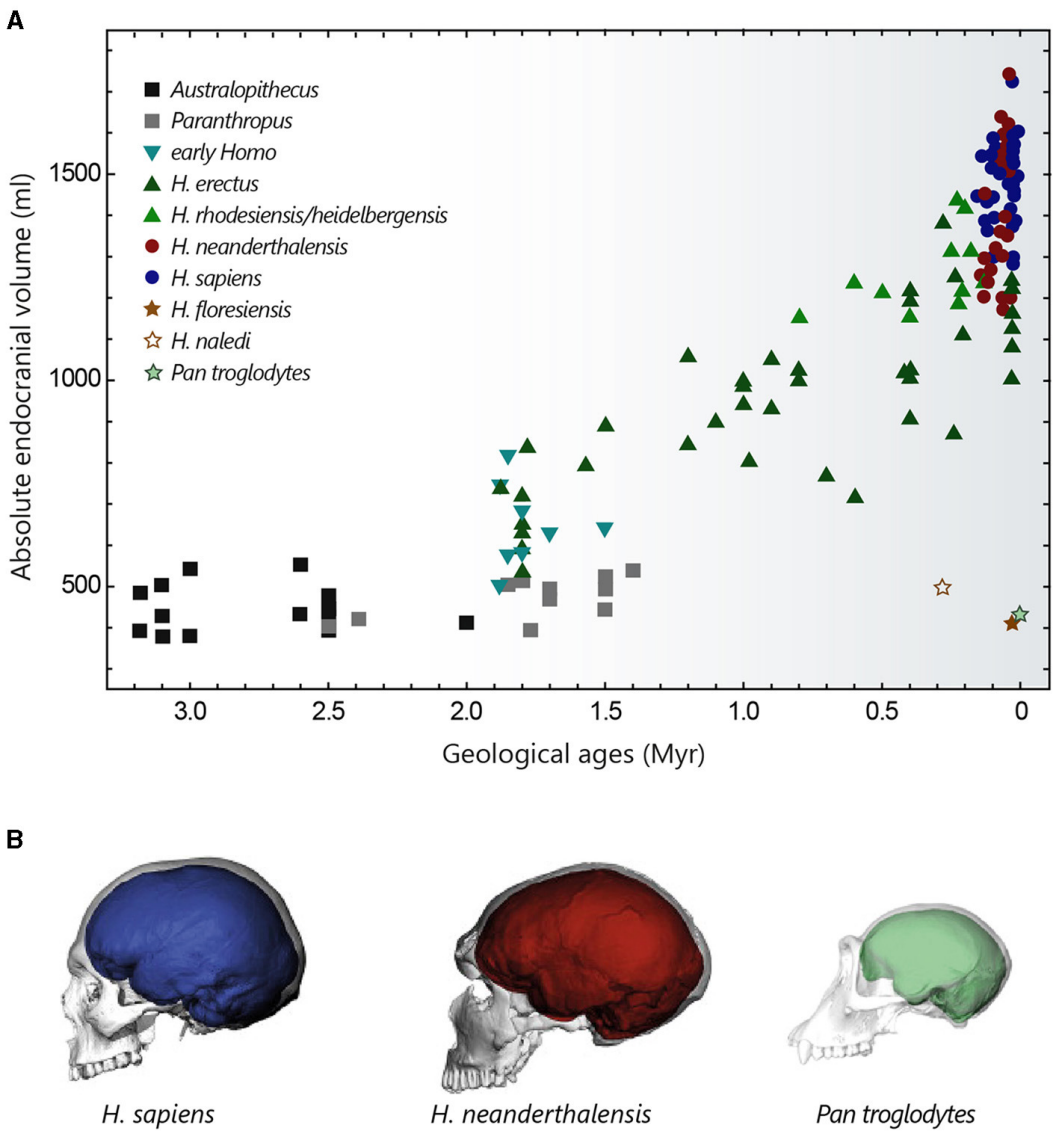
Evolution of hominin brain size. **(A)** Evolution of hominin endocranial volume over time. Adapted with permission from Hublin et al. (2015). **(B)** Differences in brain size and shape of a modern human (blue), a Neanderthal from *La Chapelle-aux-Saints* (red) and a chimpanzee (green), visualised by computer tomography. Adapted with permission from Neubauer et al. (2018, 2020).

The brain size of present-day mammals is extremely variable, ranging from <0.1 g in the Etruscan pygmy shrew to more than 9 kg in some large cetaceans (DeFelipe, 2011). In placentals, evolutionary processes have led to the emergence of large brains in several groups of species sometimes separated by a long independent phylogenetic history (Manger et al., 2013). These include a large proportion of whales (up to 9,200 g for the sperm whale) and dolphins (up to 2,900 g), elephants (up to 6,000 g), certain pinniped species (such as walruses or southern elephant seals ~1,200 g), and members of the genus *Homo* (see Figure 2). Modern *Homo sapiens*, with an average brain mass of 1,350–1,400 g (varying from 1,100 to 1,800 g), are a long way behind elephants and cetaceans, but can nevertheless claim first place among primates, since the largest brains of the great apes do not exceed 500–600 g (Tower, 1954; Jerison, 1973; Haug, 1987; Roth and Dicke, 2005; Neubauer et al., 2018). Differences in brain size in present-day *Homo sapiens* do not appear to have functional significance, since substantial variations can be observed between individuals with apparently similar intellectual abilities (DeFelipe, 2011). The idea that a larger absolute brain size should necessarily confer more complex cognitive abilities or greater behavioural flexibility is also challenged by observations that animals with similar brain weights, such as gorillas and oxen (~500 g) or elephant seals and some humans (~1,200 g), have different behavioural repertoires, or that species with relatively small brains, such as dogs (60 g), rats (2 g) and mice (0.5 g) in mammals or corvids (6–15 g) in birds, can demonstrate sophisticated behaviours (Kaminski et al., 2004; Olkowicz et al., 2016).

Another widely studied property is relative brain size. In mammals, brain size and body size are closely correlated, with a negative allometry. As a result, small species tend to have relatively larger brains in proportion to their body size than large species. In humans, for example, the brain represents around 2% of body mass, whereas this ratio can reach 10% in shrews and small rodents (Van Dongen, 1998; Roth and Dicke, 2005). It is this negative scaling relationship that led Dubois to introduce the “coefficient of cephalization,” later renamed the encephalization quotient (EQ), as a suitable measure for comparing relative brain size across species (Jerison, 1973). The EQ quantifies how much a species brain size deviates from what is expected based on its total body mass, using a standard species from the same taxonomic group as a reference. When calculating EQs in mammals, using the cat as the standard, humans emerge as the most encephalized species with an EQ of around 6–7, meaning that their brains are more than six times larger than expected. However, a high degree of encephalization is not exclusive to humans. Dolphins like the Tucuxi or the white-sided dolphin are not far behind humans with EQs around 4–5, followed by capuchin monkeys with EQs ranging from 2.4 to 4.8. Then come species like gorillas and chimpanzees (EQ = 1.5–3)—though often considered more cognitively able than capuchin monkeys (Deaner et al., 2007), elephants (EQ = 1.1–2.4), and rodents with relatively low EQ values of 0.5–1 (Jerison, 1973; Marino, 1998, 2002; Roth and Dicke, 2005; Shoshani et al., 2006). However, the ability to predict the cognitive abilities of a species from EQ values remains quite limited, mainly due to the sensitivity of this metric to the choice of the reference taxonomic group and to the value of the exponent of the power law relating brain size to body size (typically between ~0.55 and 0.8) used in the various studies (Harvey and Krebs, 1990; Marino, 1998; Charpier, 2008).

### 3.2 Brain size in hominins

The cranial capacity of hominins has undergone a significant increase over the past 3 million years, evolving from 450 cm^3^ in *Australopithecus* (a value similar to that of the great apes) to approximately 1,350–1,500 cm^3^ in *Homo sapiens* and *Homo neanderthalensis* (that went extinct ~40,000 years ago) (Figure 2A) (Jerison, 1973; Holloway, 2015; Neubauer et al., 2018). This cerebral expansion, particularly evident in *Homo* erectus (~2 million years ago), was initially closely linked to changes in body weight and then progressed more independently over the last 500,000 years (Ruff et al., 1997; Hublin et al., 2015), explaining the high EQ values of *Homo sapiens*. It is interesting to note that *Homo floresiensis*, whose remains were discovered in the Liang Bua cave on the island of Flores, represents an exception to this brain size expansion in the *Homo* lineage. Despite its ability to create sophisticated tools and perhaps to navigate, *Homo floresiensis* (dated between ~100,000 and 50,000 years ago) had unexpected morphological features such as a small stature (~1 metre) and a small endocranial volume of around 430 cm^3^ (Balzeau and Charlier, 2016; Sutikna et al., 2016). Similarly, *Homo naledi*, a new species recently discovered near Johannesburg in South Africa, which coexisted with Neanderthals and potentially with the first *Homo sapiens*, also possessed small brain capacities ranging from 465 to 610 cm^3^ (Garvin et al., 2017). These fossil discoveries are profoundly challenging our knowledge of human diversity and the long-held idea of a continuous evolution towards ever-larger human beings with ever-more-developed brains.

The increase in size of the hominin brain over the course of evolution entails an increase in its energy cost. The metabolic requirements of a human brain are considerable: accounting for 50–55% of basal metabolism at birth, this proportion peaks at over 65% at the age of 4–5 years and remains high, at around 20%, in adulthood (Clarke and Sokoloff, 1999; Kuzawa et al., 2014). It is believed that the consumption of energy-rich foods like meat and marrow, and, later, their improved digestibility through cooking (Carmody and Wrangham, 2009), have enabled mothers to allocate more energy to their foetuses during pregnancy (and their newborns during nursing) and have thus favoured the development of larger brains (Martin, 1996). Thus, the large brains of early *Homo* may have emerged as an unintended by-product of a change in maternal diet, perhaps initiated by a modification in climate and available resources. Another hypothesis suggests that a reallocation of energy to the brain may have been facilitated by a reduction in the size of other metabolically costly organs, such as the digestive system (Aiello and Wheeler, 1995). Finally, the social brain hypothesis proposes that the evolution of hominin encephalization could be the result of increasingly complex social demands in group-living species (Dunbar and Shultz, 2007).

Compared with chimpanzees and macaques (~40 and 70% brain growth *in utero*, respectively), a significant proportion of brain growth in humans occurs after birth. The size of the brain at birth is thought to be partly constrained by the anatomy of the woman's pelvis, whose dimensions are limited by biomechanical and postural factors associated with bipedalism (Hublin et al., 2015). From a size of around 400 cm^3^ at birth (~28% growth *in utero*), the brain of a young *Homo sapiens* undergoes rapid growth during the 1^st^ year, by the end of which it reaches 50% of its adult size, finally attained at the age of 5–6 years (Leigh, 2004; DeSilva and Lesnik, 2006). This perinatal phase of brain growth is accompanied by a change towards a more globular shape, typical of modern humans. This globularization process, which evolved progressively to reach the current variation between 100,000 and 35,000 years ago, is not observed in Neanderthals and chimpanzees (Figure 2B) (Bruner et al., 2003; Gunz et al., 2010; Neubauer et al., 2010, 2018).

### 3.3 The neocortical expansion

While a simple cortex is already present in the pallium of reptiles, the neocortex first appears as a complex multi-laminated structure in mammals (Nieuwenhuys, 1994; Rakic, 2009). Of modest proportion (20–48%) in small species of the three main radiations, such as platypuses, opossums, shrews and mice, it reaches considerable size in cetaceans and primates. Accounting for 75 to 84% (depending on the study) of the total mass or volume of the brain, the human neocortex is proportionally one of the largest among mammals. Humans are however closely followed (and sometimes equalled) by most odontocete cetaceans (~72%), as well as several species of monkeys and apes, such as macaques (70–76%), grivets (79%), or chimpanzees (70–73%) (Pirlot and Nelson, 1978; Stephan et al., 1981; Hofman, 1988; Rilling and Insel, 1999; Manger, 2006). Within the neocortex, a pronounced enlargement of the frontal lobe, thought to be involved in higher cognitive functions, has long been regarded as a hallmark of human evolution. Brodmann (1912), shortly after completing his famous cytoarchitectural map of the cerebral cortex, published a comparative study of the frontal cortex surface in primates, demonstrating a progressive increase from prosimians to humans. While more recent studies have indeed demonstrated an increase in the absolute size of the frontal cortex in humans (Semendeferi et al., 1997, 2002; Bush and Allman, 2004), this expansion does not seem to significantly differ from what would be expected from a great ape with a human-sized brain (Semendeferi et al., 2002; Barton and Venditti, 2013).

Neocortex thickness generally correlates positively with brain size (Hutsler et al., 2005; Balaram and Kaas, 2014). However, this correlation does not uniformly apply to all taxa, as evidenced by the typically thin cortex (<2 mm) of cetaceans (Ridgway and Brownson, 1984). The average variation in cortical thickness between species (between 0.4 and 2.8 mm), which is comparable to the variability found between cortical areas in the same animal, remains relatively modest compared to the variation in overall brain size. This suggests that the expansion of the neocortex in large-brained mammals is mainly the result of an increase in its surface area (rather than its thickness), often resulting in the formation of convolutions, particularly visible in large primates and cetaceans (Hofman, 1985, 1988; DeFelipe, 2011 for review). In primates, a gradual increase in neocortical thickness is observed from primary to more integrative sensory areas, a trend seemingly absent in motor and frontal association cortices (Wagstyl et al., 2020). Finally, the relative thickness of cortical layers also varies between species, with supragranular layers being proportionally thicker in primates than in carnivores and rodents, while infragranular layers show an inverted profile (Hutsler et al., 2005).

## 4 Intrinsic organisation of the neocortex

### 4.1 Areas and columns

The mammalian neocortex is typically subdivided into six layers defined by vertical differences in the size, shape, or density of neurons (Brodmann, 1909). There are, however, variations in the number, thickness or overall cytoarchitectonic organisation of the layers across the cortical mantle (Kaas, 1987; DeFelipe, 2011), which have formed the basis of its subdivision into distinct regions.

The neocortex is classically regarded as a complex mosaic of anatomically and functionally specialised areas whose number increases with brain size (Brodmann, 1909). From about 10 to 15 cytoarchitectonic subdivisions mainly dedicated to sensory processing (primary somatosensory, visual, and auditory areas) and motor functions (although controversy remains over a clear separation of somatosensory and motor regions in some early mammals) in small-brained species (Krubitzer et al., 1995; Catania et al., 1999; Kaas, 2011), their number could approach 200 in humans (Glasser et al., 2016). In large-brained species, primary sensory and motor fields subdivide—more than 10 to 20 areas identified for the single visual cortex in cats and monkeys—, change in size and relative position and become separated by the inclusion of associative areas notably in the frontal and temporo-parietal regions (Nieuwenhuys, 1994; Northcutt and Kaas, 1995). The small amount of cortical territory devoted to multimodal or association areas in monotremes suggests that unimodal sensory fields could constitute the core of the prototypical plan for neocortical organisation in mammals (Krubitzer et al., 1995). The developmental mechanisms responsible for this elaborate cortical parcellation have been debated as to whether the structural differences between areas are induced in a homogeneous population of cortical neurons by the patterned activity of thalamocortical projections, or whether the formation of the neocortical map is already genetically determined in the neural progenitors of the embryonic ventricular zone. In this later view, which seems to be gaining consensus, areas in the cortical plate would attract appropriate inputs rather than being specified by them. Activity-dependent mechanisms would then play an influential role at later stages in refining existing synaptic connexions (Rakic, 2002).

The predominance of vertical over horizontal connexions in his anatomical reconstructions of rodent cortical neurons, led Lorente de Nó (1938) to suggest in the 1930s that cortical areas were composed of multiple 'elementary units' of information processing taking the form of vertical bands of interconnected neurons. Almost 20 years later, Mountcastle obtained persuasive evidence of a columnar segregation of sensory modalities in the cat somatosensory cortex by showing that neurons recorded along different vertical microelectrode tracks responded either to superficial or deep cutaneous stimulation. He introduced the term 'cortical column', assigned them an average width of ~0.5 mm and demonstrated that the different functional columns were intermingled in the manner of a mosaic (Mountcastle, 1957). Columnar organisation formed by groups of neurons activated more strongly by stimulation of one of the two eyes (ocular dominance column) or by stimuli having a common receptive field axis orientation (orientation columns) were later discovered in the cat primary visual cortex (Hubel and Wiesel, 1963, 1969). Comparing data obtained in cats and macaques, Hubel and Wiesel (1963, 1974) found that similar variations in orientation tuning were obtained with smaller electrode advances in monkeys, suggesting thinner columns or less defined borders in this species. Variations in the width of ocular dominance columns (from 200 to 800 μm) were also reported in subsequent studies on different primate species including humans (Bugbee and Goldman-Rakic, 1983; Horton and Adams, 2005), finally leading to the introduction of a new entity, the minicolumn, whose iteration and lateral combination through short-range horizontal connexions would form the basis of functional columns. Developmentally, minicolumns reflect the radial migration of neurons from the proliferative ventricular zone into narrow (30–50 μm) translaminar chain of cells separated by neuropil (Buxhoeveden and Casanova, 2002; Rakic, 2002). According to the “radial unit hypothesis,” the surface expansion of the neocortex during mammalian evolution (by ~10,000 times from shrews to the largest cetaceans; Hofman, 1985; Manger, 2006), with no comparable variation in its thickness, could result from a change in the genetic mechanisms that control the timing and/or mode of cell division in the ventricular zone, leading to an increase in the pool of founder cells at the origin of radial columns (Rakic, 1995; Chenn and Walsh, 2002).

Anatomical and functional evidence for a modular organisation of the neocortex has been obtained in a wide range of species from different mammalian radiations. Alternating bands of corticocortical projections related to monoaural or binaural responses are observed in the cat primary auditory cortex (Imig and Adrián, 1977), and patchy arrangements of axon terminal fields are apparent in the auditory area of the short-beaked echidna (Dann and Buhl, 1995). An additional example is the discrete architectonic units, known as “barrels,” formed by neurons preferentially activated by the same facial whisker in the primary somatosensory cortex of rodents and several other mammals with whiskers (Woolsey and Van der Loos, 1970). In the platypus somatosensory area, regions where neurons respond only to cutaneous stimulation of the bill are separated from regions where neurons process both tactile and electrical inputs (Krubitzer et al., 1995). Most frequently observed in primary sensory systems, the presence of columns is also attested in primary motor and association cortices (Bugbee and Goldman-Rakic, 1983; Amirikian and Georgopoulos, 2003). The existence of relatively similar organisational patterns in various areas and species led to idea that modular units could represent a fundamental principle of cortical function in mammals, important for perception, cognition and memory (Eccles, 1981; Mountcastle, 1997). In this context, it is expected that the subdivision of cortical regions into iterated computational units capable of operating in parallel should increase the number of possible spatio-temporal combinations of activity, and hence the processing capacities of large brains. However, the concept of cortical columns has also been contested based on an apparent intra- and inter-species inhomogeneity in size, shape, and expression without obvious differences in cortical function. For example, ocular dominance columns are well defined in Old World monkeys and remain rudimentary in most New World monkeys (Hendrickson et al., 1978; Adams and Horton, 2003), despite similar visual abilities. Similarly, barrels are not found in all the marsupial species that possess whiskers, and some rodents, like the chinchilla, have barrel fields without engaging in whisking behaviour (Purves et al., 1992). These findings raise the possibility that cortical modules may have emerged in different forms during areal specification in mammals, without acquiring an obvious function in all species (Horton and Adams, 2005).

### 4.2 Neurons and synapses in numbers

The mammalian neocortex contains approximately 15–25% of the total number of brain neurons (Azevedo et al., 2009; Herculano-Houzel, 2012). Initial assumptions that cortical columns were composed of a constant number of neurons in all mammals (Rockel et al., 1980) have been challenged by subsequent studies showing, using stereological and non-stereological counting methods, variations in the density of neurons between species, areas and layers (DeFelipe et al., 2002; Herculano-Houzel et al., 2008). Neuronal density in the neocortex generally tends to be inversely correlated with brain volume, with different scaling rules applying to different orders of mammals. Thus, for a similar increase in neocortex mass, the corresponding decrease in neuronal density seems to be less pronounced in primates than in other placental mammals (Haug, 1987; Manger, 2006; Herculano-Houzel, 2012; Herculano-Houzel et al., 2014), and marsupials are reported to have fewer neurons than placentals of equivalent brain size (Haug, 1987; Seelke et al., 2014). The total number of cortical neurons in the human brain (12–16 billion) therefore exceeds that measured in other large-brained species such as whales or elephants (6–11 billion), but this number aligns with expectations for a primate with a human-sized brain (Haug, 1987; Azevedo et al., 2009; Herculano-Houzel et al., 2014). However, Pinson and colleagues recently discovered that expression of the modern human variant of transkelotase-like protein 1 (hTKTL1)—but not of the Neanderthal variant (which differs by a single amino acid substitution)—in the embryonic mouse neocortex can increase the abundance of a specific type of basal progenitors and promote neuron production, especially in the frontal lobe. These findings suggest that, even within primates, species with similar brain sizes, such as *Homo sapiens* and Neanderthals, may exhibit variations in the number of neurons (Pinson et al., 2022).

Differences in counting methods, variations in age and number of samples, or in the amount of cortical volume examined make it difficult to compare calculations of synaptic density between laboratories (DeFelipe et al., 2002). Nevertheless, most studies agree that the mean synaptic density in the adult (defined as the total number of excitatory and inhibitory synapses per unit volume of cortical tissue) do vary across species (between ~250 and 1,000 million/mm^3^), but relatively independently of brain size (see DeFelipe et al., 2002; Karbowski, 2014 for reviews). For instance, a recent comparative study conducted in 25 primate species (including humans) found relatively constant synaptic densities in the primary visual and inferior temporal cortex of the different animals (~256 million/mm^3^), varying by only 1.9-fold despite brain weights differing by about 500-fold (Sherwood et al., 2020). Although a certain percentage of synapses continue to be remodelled in adulthood, synaptic density in the adult neocortex is globally stationary. This period of stable synaptogenesis is preceded by major changes in the rate of synapse production, which is particularly high during the perinatal period. The duration of this massive increase in synapse density around birth varies widely between mammals, ranging from 2 weeks in rats, 1 month in cats, 4 months in macaques, to around 3 years in humans (Bourgeois, 2008). The number of synapses is then maintained at a maximum until puberty (which is delayed in primates compared with rats and cats), during which synaptic density decreases markedly to levels comparable to those observed in adults (Huttenlocher, 1979; Bourgeois and Rakic, 1993; Bourgeois, 2008; Elston and Fujita, 2014). The maturation period for synaptic architecture is therefore considerably lengthened in primates, suggesting that the sensory environment could play an important role in the configuration and refinement of cortical circuits.

The number of synapses per neuron, usually estimated by dividing the synaptic density by the neuronal density in a given layer, positively correlates with brain volume. In primates, the overall number of synapses per neuron (including both excitatory and inhibitory cells) in the inferior temporal cortex is thus higher in humans (~4,850 synapses/neuron) than in gorillas (~3,550 synapses/neuron), chimpanzees (~2,885 synapses/neuron), and macaques (~2,160 synapses/neuron) (Sherwood et al., 2020). However, this positive scaling does not universally apply to all species, as sensory cortex neurons in mice are reported to have more synapses than in rats (DeFelipe et al., 2002). The ratio of synapse density to neuron density remains a relatively coarse measure of connectivity because it does not differentiate between different types of neurons and overlooks the fact that dendrites, particularly those of pyramidal neurons, generally span several layers. A more accurate estimate of the number of synapses received by a given neuron could perhaps be obtained by quantifying the spine density along small dendritic segments (assuming that each dendritic spine is contacted by at least one synaptic input) and extrapolating these measurements to a cumulative number of spines, considering the length of the different dendritic compartments. Such analyses revealed that the density of spines on pyramidal neurons from the supragranular layers of the temporal cortex is higher in humans than in macaques (1.35 times), marmosets (1.9 times), or mice (1.3 times) (Elston et al., 2001; Benavides-Piccione et al., 2002). Based on these calculations and measures of total dendritic length, the total number of synapses received by a human temporal cortex L2/3 pyramidal cell has been estimated to be around 20,000 (Eyal et al., 2018).

### 4.3 Constituent cell types and functional microcircuit organisation

Cortical circuit computations rely on the dialogic interaction of two main classes of neurons: the spiny glutamatergic excitatory neurons (comprising pyramidal and stellate cells), processing and transmitting information within and/or outside the neocortex, and the smooth or sparsely spiny GABAergic inhibitory interneurons, which finely regulate synaptic activity of local populations of excitatory neurons, shaping network dynamics.

**Figure 3:**
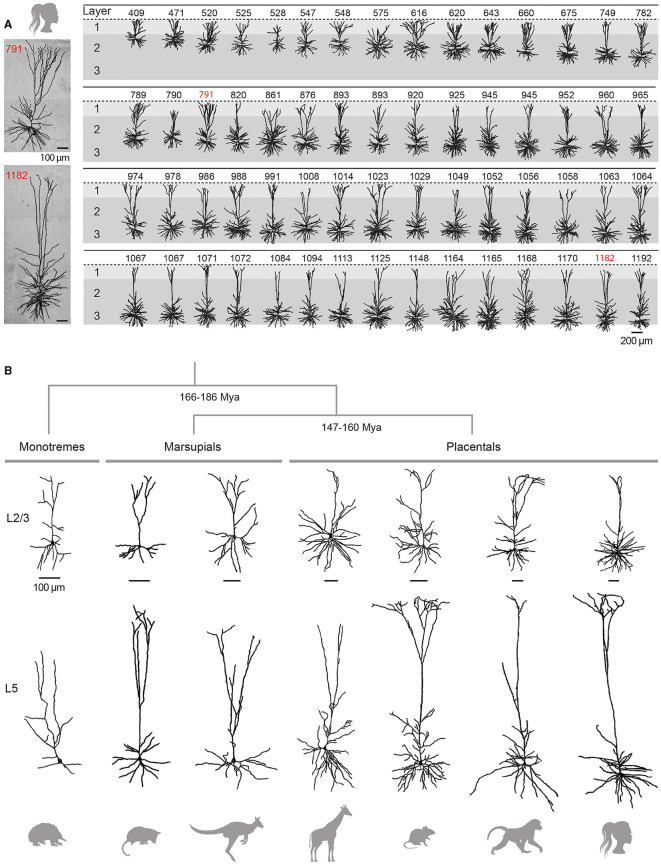
Variability and evolution of pyramidal neuron morphology in mammals. **(A)** Database of reconstructed human temporal cortex L2/3 neurons, classified according to their somatic depth, indicated in μm at the top of each cell. Two examples of cells whose soma was located 791 **(top)** and 1182 **(bottom)** μm from the cortical surface are enlarged on the left. Adapted with permission from Deitcher et al. (2017). **(B)** Examples of supragranular (L2/3, top) and infragranular (L5, bottom) pyramidal neurons from the visual (L2/3) and motor (L5) cortices of the short-beaked echidna, the primary motor cortex of Benett's wallaby, the sensorimotor cortex of the American opossum, the primary motor cortex of the northern giraffe, the primary somatosensory cortex of the Sprague-Dawley rat, the primary motor cortex of the chacma baboon and the human temporal cortex. Adapted from Dann and Buhl (1995) (echidna L2/3); Hassiotis and Ashwell (2003) (echidna L5), Jacobs et al. (2018) (wallaby, baboon, giraffe), Boyer et al. (2022) (rat L2/3), Mahon and Charpier (2012) (rat L5), Deitcher et al. (2017) (human L2/3), and Kalmbach et al. (2021) (human L5). Neurons were redrawn from the sources mentioned. Scale bars (100 μm) are species-specific and apply to supra- and infragranular neurons. Estimates for monotreme-therian divergence and marsupial-placental separation are taken from Bininda-Emonds et al. (2007) and Phillips et al. (2009).

The following sections will primarily address the structural characteristics of the pyramidal neuron, accounting for ~70–80% of the total population of neocortical neurons in placental mammals. Qualified as the “psychic cells” of the brain by Santiago Ramón y Cajal, pyramidal neurons are distributed across all cortical layers (except L1, where they still extend dendrites), and are regarded as the cornerstone of the cortical microcircuitry. The typical eutherian mammalian pyramidal neuron is distinguished by its prominent apical dendrite, radially oriented towards the pia, and its skirt of basal dendrites radiating from the soma (Figure 3). Pyramidal cells can be broadly classified as intratelencephalic (IT) or extratelencephalic (ET), depending on whether their long-range axons are confined to telencephalic structures (such as the neocortex, striatum, or claustrum) or whether they additionally establish connexions with brain structures outside the telencephalon (thalamus, tectum, pons, spinal cord). IT neurons are distributed throughout layers 2 to 6, while ET cells are confined to the deeper layers 5–6 (Harris and Shepherd, 2015; Baker et al., 2018a for reviews). IT neurons are the sole source of interhemispheric connexions, conveyed through the anterior commissure in monotremes and marsupials, as well as through the corpus callosum in eutherians (Suárez et al., 2018). Spiny stellate cells, lacking a prominent apical dendrite and instead featuring a star-like dendritic arbour, are predominantly localised in L4 of primary sensory cortices. Below I present an overview of the organisation of cortical circuits, outlining the main classes of neurons and their input-output connectivity patterns. This description is mainly based on findings obtained in eutherian mammals (in particular rodents, cats, and monkeys), with attempts to draw comparisons with the monotreme and marsupial literature where feasible. I will not cover here the properties of cortical astrocytes, which are now recognised as key contributors to various neuronal functions, including synaptic transmission, energy metabolism, and ion homeostasis. However, investigating their diversity and morpho-functional features within the main mammalian groups represents a promising direction for future research, given the reported variations in the number or size of protoplasmic astrocyte processes between humans and rodents, as well as the specific presence of certain astrocyte types in primates (see Oberheim Bush and Nedergaard, 2017 for review).

#### 4.3.1 Upper-layer pyramidal neurons in eutherians

L2/3 pyramidal neurons, a subset of IT neurons, play a pivotal role in intra-cortical information processing through their local and long-range corticocortical connexions. These neurons receive inputs from specific thalamic nuclei on their basal dendrites, either directly or *via* ascending projections from L4 in sensory cortices or from upper L5 in cortices lacking a distinct granular layer, while non-specific thalamocortical and distant cortical inputs mainly terminate on their tuft branches in L1. L2/3 pyramidal neurons make numerous reciprocal connexions within their home column, mostly on upper basal and apical oblique dendrites. Locally, L2/3 ITs send prominent descending axonal projections to L5 pyramidal cells (Weiler et al., 2008; Lefort et al., 2009; Petreanu et al., 2009; Harris and Shepherd, 2015). This robust output to L5 has been identified as an essential feature of neocortical microcircuitry, preserved in most regions and species (Thomson and Lamy, 2007; Weiler et al., 2008; Hooks et al., 2011). Long-range axons of supragranular pyramids establish connexions with ipsi- and contralateral cortical regions and the striatum (Petreanu et al., 2007; Anderson et al., 2010; Pidoux et al., 2011a).

L2/3 pyramidal neurons show substantial regional and inter-species variation in their dendritic architecture. In primates, L2/3 ITs from higher integrative frontal cortices generally display a more extensive and branched basal dendritic tree than their counterparts from primary and secondary sensory areas (Elston and Rosa, 1998; Elston et al., 2001; Jacobs et al., 2001; Gilman et al., 2017; Galakhova et al., 2022). A similar size increase in basal arborization along the caudal-rostral axis has been reported in elephants (Jacobs et al., 2011) and rodents (Benavides-Piccione et al., 2006; Elston et al., 2006), although with less pronounced regional differences (Mohan et al., 2015; Gilman et al., 2017). A cross-species comparison indicates that upper-layer neurons in frontotemporal regions of macaques and humans have a larger total dendritic length (on average 1.5-fold for macaques and 3-fold for humans) and greater branching complexity than homologous neurons in mice (Mohan et al., 2015; Gilman et al., 2017), whereas such differences are not observed in primary visual cortices (Gilman et al., 2017). When comparing mammals with large brains, the length of the basilar dendritic arborization in elephants is reported to be slightly longer in both frontal cortex (~7%) and occipital cortex (~3%) than in humans, despite a lower degree of branching in the former species (Jacobs et al., 2011).

Traditionally considered as a homogeneous cell population, there is increasing evidence for depth-dependent differences in the morpho-functional properties of L2/3 pyramidal cells in rodents (Brecht et al., 2003; Lübke et al., 2003; Staiger et al., 2015) and for the presence of neuronal subclasses distinguished by the expansion of their apical dendritic arborization, as observed in the rat medial prefrontal cortex (van Aerde and Feldmeyer, 2015). This morphological diversity of L2/3 pyramidal neurons appears to be even more pronounced in humans, with a significant increase in the extent of the horizontal field span of the apical tree, length of the basal arborization and mean radius of the cell body with increasing distance from the pia (Figure 3A) (Deitcher et al., 2017; Berg et al., 2021). In addition, recent RNA-sequencing studies have revealed the existence of two additional transcriptomically defined subtypes of pyramidal neurons in deep human L3, apparently absent in mice (Hodge et al., 2019; Berg et al., 2021).

The vast majority (70–95%) of excitatory synaptic inputs target the dendritic spines of neocortical pyramidal neurons (Nieuwenhuys, 1994). The elongated dendritic trees in multimodal and association cortices often correlates with an increase in spine density, particularly evident in frontal regions in primates (Elston et al., 2001; Jacobs et al., 2001). However, this trend does not hold true for all species. For examples, pyramidal neurons in the prefrontal cortex of the marmoset have fewer branches and spines than those in the temporal lobe (Elston et al., 2001), and the density of spines in the macaque prefrontal cortex does not exceed that observed in different regions of the mouse neocortex (Gilman et al., 2017). The length of dendritic spines (~1.3 μm combining neck and spine head) does not seem to significantly change with cortical size (Karbowski, 2014), despite a slight tendency for spines in the human temporal cortex to have longer necks (0.9 vs. 0.7 μm) and larger heads (0.6 vs. 0.4 μm^2^) compared to those in mice (Benavides-Piccione et al., 2002). Finally, in line with the idea that a dynamic balance between excitatory and inhibitory activities is a fundamental principle of cortical circuit function in physiological conditions, the ratio between excitatory (~70–90%) and inhibitory (~10–30%) synapses appears globally conserved in mammals, although laminar-dependent differences within and between species can be observed (for reviews DeFelipe et al., 2002; Karbowski, 2014).

#### 4.3.2 Deep-layer pyramidal neurons in eutherians

Infragranular pyramidal neurons integrate inputs form virtually all neocortical layers thanks to their elongated apical dendrite and radiating basilar arborization. They in turn significantly influence cortical and subcortical operations through local connectivity and long-range output projections. L2/3 inputs to L5 cells are distributed along the dendritic tree, mainly on tuft branches, apical oblique and basal dendrites. Afferents from L4 mostly terminate on basal dendrites, which also receive axons from primary relay thalamic nuclei. Additionally, L5 pyramidal cells receive thalamocortical projections from higher-order thalamic nuclei on their apical tuft in L1 and basal dendrites (Weiler et al., 2008; Petreanu et al., 2009; Hooks et al., 2011). L5 ITs establish many reciprocal connexions and provide synaptic inputs to L5 ETs. Connexions from ETs to ITs are less numerous (Morishima and Kawaguchi, 2006; Brown and Hestrin, 2009; Kiritani et al., 2012); ET intracortical projections mainly contributing to inter-areal communications (Nelson et al., 2013; Ueta et al., 2013; Harris and Shepherd, 2015). Most of inputs to L6 ITs in sensory cortices originate from local deep-layer neurons, while L6 ETs are primarily innervated by axons from higher-order cortical areas (Zhang and Deschênes, 1998; Mercer et al., 2005; Feldmeyer, 2012; Vélez-Fort et al., 2014).

Although IT and ET neurons are distributed across L5 and L6, they display laminar-dependent projection patterns. For instance, corticostriatal neurons projecting to ipsilateral and/or contralateral striatum are found throughout L5 (Anderson et al., 2010; Pidoux et al., 2011a), while corticospinal neurons seem to be confined to the deeper part of L5 (Anderson et al., 2010; Suter et al., 2013), and corticothalamic neurons predominate in L6 (Bourassa and Deschênes, 1995). Corticothalamic neurons from sensory areas typically project back to their primary relay thalamic nuclei, but those located in the deeper part of L6 may also project to higher-order thalamic nuclei (Bourassa and Deschênes, 1995; Chevee et al., 2018).

The two subclasses of infragranular pyramidal neurons differ in their apical dendritic architecture, with great variability existing within each subpopulation in all species (for review, see Baker et al., 2018a). Traditionally, L5 ET neurons possess a more complex apical dendritic arborization with numerous branches and a crown-shaped tuft that unfolds close to the pial surface (Figure 3B), whereas the apical dendritic tuft of IT neurons is more restricted with fewer side branches (Hattox and Nelson, 2007; Ramaswamy and Markram, 2015; Kalmbach et al., 2021). Morphological heterogeneity is also present within L6; L6 ETs exhibit a relatively compact apical dendritic arborization predominantly terminating in narrow tufts in L4, while corticocortical L6 neurons extend an untufted or sparsely tufted apical dendrite, rarely extending beyond L4-L5 border. By contrast, the apical dendrite of the corticoclaustral IT neurons in L6 can reach the lower boundary of L1 (Katz, 1987; Zhang and Deschênes, 1997; Kumar and Ohana, 2008; Oberlaender et al., 2012; Yang et al., 2022). Consistent with inter-areal variations observed in superficial layers, infragranular pyramidal cells from higher processing areas possess a more elaborate basal dendritic arborization in the macaque (Elston and Rosa, 2000). Despite the difficulty of unambiguously identifying ET neurons in humans (based on their axonal projections), a transcriptomic cell class sharing multiple distinctive marker genes and morphological attributes with the murine ET neuron subtype (Tasic et al., 2016) has been identified in different regions of the human neocortex, despite a relative lower abundance as compared to monkeys and rodents (Hodge et al., 2019; Bakken et al., 2021; Kalmbach et al., 2021).

Certain subpopulations of ET neurons, with morphological features that deviate from the archetypal pyramidal neuron, are endemic to certain cortical areas and species. For instance, the corticospinal gigantopyramidal neuron (Betz cell in primates), with its very large cell body and extensive basilar dendrites, is exclusively found in the primary motor cortex of carnivores and primates (reviewed in Jacobs et al., 2018). The same applies to the large von Economo neuron, which is characterised by its spindle-shaped soma and thick, poorly branched apical and basal dendrites. Initially described in human anterior cingulate and frontoinsular cortices, they were first considered to be specifically human and identified as particularly prone to early loss and morphological alterations in various neuropsychiatric disorders (reviewed in Butti et al., 2013). Their existence, with similar cortical distributions and in fairly comparable numbers, was subsequently demonstrated in other large-brained species such as great apes, elephants and certain cetaceans, although their presence in non-primate mammals is still debated (Butti et al., 2013; Banovac et al., 2019).

#### 4.3.3 Pyramidal neurons in marsupials and monotremes

Pyramidal neurons have been clearly identified in both marsupials and monotremes (Figure 3B), but a detailed analysis of their dendritic architecture and synaptic connectivity is still lacking (marsupials: Walsh and Ebner, 1970; monotremes: Dann and Buhl, 1995; Tyler et al., 1998; Elston et al., 1999; Hassiotis and Ashwell, 2003; Hassiotis et al., 2005; Jacobs et al., 2018).

Several key features of the eutherian pyramidal cell are retained in marsupials, including their presence throughout layers 2 to 6, an upward-projecting apical dendrite, elaborate basilar dendritic arborization, and recurrent excitatory synaptic connexions. Some variations, such as the more common bifurcation of apical dendrites into daughter branches, were however observed in wallabies, quokkas, and opossums, but not in dunnarts (Walsh and Ebner, 1970; Tyler et al., 1998; Jacobs et al., 2018). Our knowledge on neuronal classes in monotremes is still limited but the few existing studies suggest that pyramidal neurons in the short-beaked echidna represent a smaller proportion (35–50%) of the total population of cortical neurons compared to therian species. In addition, a substantial number of these pyramidal neurons (30–40%) display atypical attributes such as apical dendrites lacking a terminal bouquet or branching close to the soma, and poorly developed basal dendritic skirts. Monotreme pyramidal cells also appear to have a lower density of spines on apical and/or basal dendrites. However, the morphology of the different types of non-pyramidal neurons (spiny stellate cells and inhibitory interneurons) is very similar in monotremes, marsupials, and placentals. These observations led Hassiotis and colleagues to put forward the hypothesis that pyramidal and non-pyramidal neurons may have emerged as distinct morphological entities in the first mammals, while the entire set of typical pyramidal cell features would have appeared shortly after the split with the prototherian lineage, around 180 million years ago (Hassiotis and Ashwell, 2003; Hassiotis et al., 2005).

In summary, the morphology of eutherian pyramidal neurons appears more diverse than previously thought, even within a single cortical area of a given species (see, for example, Figure 3A). Furthermore, it is interesting to note that the early bifurcation of apical dendrites in marsupials and monotremes is an anatomical feature that is also commonly observed in some placental species, such as hedgehogs or elephants (Valverde and Facal-Valverde, 1986; Jacobs et al., 2011). Thus, the canonical and non-canonical aspects of pyramidal cells seem to have been relatively well preserved during cortical evolution; a better understanding of neuronal properties in clades close to the root of the mammalian phylogenetic tree will help to clarify whether morphological heterogeneity is indeed greater in these species and whether it could translate into different cortical functioning.

#### 4.3.4 GABAergic interneurons

Neocortical GABAergic interneurons constitute a highly heterogeneous set of cells that differ in their morphology, input-output connectivity, intrinsic electrophysiology, and expression of molecular markers such as calcium-binding proteins and neuropeptides. Although a consensus classification of interneurons is still debated (Ratliff and Batista-Brito, 2020), available data suggest a grouping into four main subclasses based on distinct immunohistochemical profiles. These include interneurons expressing the calcium-binding protein parvalbumin (PV), the neuropeptide somatostatin (Sst), and the ionotropic serotonin receptor 5HT3a (5HT3aR), with the 5HT3aR group being further divided into two subgroups based on vasoactive intestinal peptide (VIP) expression. Each of these molecularly identified subclasses encompasses several interneuron types, primarily defined by specific morpho-functional properties (Ascoli et al., 2008; Rudy et al., 2011; Tremblay et al., 2016). The different interneurons target preferential subcellular domains on neighbouring pyramidal neurons, presumably mediating specific functions within the cortical microcircuit. For instance, within the PV-positive group, fast-spiking basket cells mostly synapse on somatic and proximal dendritic regions, while chandelier (axo-axonic) cells innervate the axonal initial segment. In addition to their local axonal arborization, Sst-expressing Martinotti interneurons project to superficial layers, where they inhibit apical tuft dendrites. The vast majority of 5HT3aR/VIP cells located in superficial cortical layers have a bipolar-like dendritic morphology and vertically extending axons that exert a translaminar inhibitory influence on the basal dendrites of pyramidal cells, although they preferentially form synapses onto other interneurons. Finally, the 5HT3aR/non-VIP neurons mainly target dendrites of local pyramids via their dense axonal plexus (neurogliaform cells) or provide a perisomatic inhibition (5HT3aR/non-VIP, cholecystokinine-positive cells) (for comprehensive reviews, see Markram et al., 2004; Tremblay et al., 2016; Lourenço et al., 2020). Recent work combining analysis of transcriptomes, intrinsic electrical properties, and morphological features of GABAergic interneurons from the mouse visual cortex, has further refined the interneuron classification system, revealing an even greater diversity of cell types (Tasic et al., 2018; Gouwens et al., 2020).

Following Ramón y Cajal (1917)'s belief that “the functional superiority of the human brain is intimately bound up with the prodigious abundance and the unusual wealth of forms of the so-called neurons with short axon”, neuroanatomists have long speculated about an increased diversity of inhibitory interneurons in the primate brain (Nieuwenhuys, 1994). There is now growing evidence that the main subclasses of interneurons are shared by the three mammalian lineages, with substantial similarities in their anatomical and physiological properties. For instance, key features of neurogliaform interneurons described in rodents, including genetic marker expression, morphological characteristics, firing patterns, and neuromodulation, are conserved in macaques and humans (rodent: Tamás et al., 2003; rodent and primate: Oláh et al., 2007; Povysheva et al., 2007; Poorthuis et al., 2018), despite a seemingly higher intrinsic excitability in primates (Povysheva et al., 2007; Poorthuis et al., 2018). Species vary, however, in the overall percentage and laminar distribution of interneurons, as well as in the relative proportion of different subtypes (Fairén and Regidor, 1984; Kawaguchi and Kubota, 1997; Tyler et al., 1998; Hof et al., 1999; Hassiotis and Ashwell, 2003; Zaitsev et al., 2005; Krienen et al., 2020). Immunochemical and transcriptomic studies estimate the percentage of inhibitory interneurons in the neocortex to be approximatively 15% in rodents, compared to an average of 20–30% in humans, monkeys, and cetaceans (Glezer et al., 1993; DeFelipe et al., 2002; DŽaja et al., 2014; Krienen et al., 2020; Bakken et al., 2021).

Some interneurons subtypes appear specific to certain species. Originally described by Ramón y Cajal in the human neocortex, calbindin-positive double bouquet cells, characterised by long-descending bundles of highly varicose axonal collaterals (the so-called “horse-tail” neurons), are mostly found in primates and, to a lesser extent, in carnivores (Ballesteros-Yáñez et al., 2005; DeFelipe et al., 2006). Even though neurons with radially oriented dendritic and/or axonal arborizations that resemble horse-tail neurons have been described in other placental and marsupial mammals (Somogyi and Cowey, 1984; Kawaguchi, 1995; Tyler et al., 1998), they display a less laterally confined axonal plexus with fewer varicosities in these species. Finally, a recent study has identified a group of L1 interneurons in humans distinguished by a specific immunochemical profile and anatomical features, including a compact bushy axonal arborization and large rosehip-shaped axonal boutons. These “rosehip cells,” which predominantly target the apical dendrites of pyramidal cells, are well positioned to modulate distal dendritic computation (Boldog et al., 2018).

#### 4.3.5 The sensory cortical microcircuit

In the late 1980s, the observation of similarities in the composition and distribution of cortical neurons between species gave rise to the idea that a common canonical microcircuit might exist in mammals (Douglas et al., 1989; Douglas and Martin, 2004). Although the multidimensional nature of connectivity schemes rules out the possibility of a single cortical circuit, comparative analysis of the data accumulated in placental mammals does suggest the presence of shared principles of organisation and function (see Silberberg et al., 2002; Douglas and Martin, 2004; Thomson and Lamy, 2007; Harris and Shepherd, 2015 for reviews). The key stages in the integration of sensory input across cortical layers are described below, drawing primarily on research in rodents and cats.

Sensory inputs from primary thalamic relay nuclei are routed to the cortex *via* modality-specific channels. These thalamocortical projections predominantly terminate on L4, but also at the boundary between L5 and L6. For their part, axons from higher-order thalamic nuclei primarily target L1 and L5, while avoiding L4 neurons (Thomson and Lamy, 2007; Petreanu et al., 2009; Constantinople and Bruno, 2013; Harris and Shepherd, 2015). L4 is considered as the main thalamorecipient layer and the starting point of intracortical sensory processing. L4 principal neurons comprise two types of glutamatergic cells: spiny stellate and star pyramidal cells, which have broadly similar functional properties but vary in proportion between areas and species. In rodents, spiny stellate cells are abundant in the primary somatosensory cortex, but rare in the visual cortex (Peters and Kara, 1985; Feldmeyer, 2012). Conversely, these cells are prevalent in the primary visual cortex of cats, monkeys and humans, while remaining sparse and randomly distributed among pyramidal cells in the auditory cortex (Lund, 1984; Meyer et al., 1989; Smith and Populin, 2001; Nassi and Callaway, 2009). Stellate cells are also found in the dunnart (Tyler et al., 1998) and in the echidna, where they exhibit a relatively wider distribution spanning L3 and L5 (Hassiotis and Ashwell, 2003).

L4 spiny neurons receive dense intracortical excitatory inputs (Schubert et al., 2003; Thomson and Lamy, 2007; Lefort et al., 2009), which likely enhance the gain and duration of thalamocortical responses to ensure a robust representation of sensory information (Lien and Scanziani, 2013; Li L. Y. et al., 2013; Li Y. T. et al., 2013). In the primary visual cortex of the cat, most of the glutamatergic connexions onto L4 spiny neurons originate from other L4 neurons and L6 pyramidal cells, whereas thalamocortical afferents account for only about 6% of the total synaptic contacts in L4 (Ahmed et al., 1994). The proportion of thalamocortical inputs to spiny neurons is larger in the mouse somatosensory cortex, reaching an average of ~16% of excitatory inputs (Benshalom and White, 1986). Thalamic projections also terminate on L4 GABAergic interneurons, resulting in powerful feedforward inhibition (Cruikshank et al., 2007) that timely regulates the firing of excitatory cells and contributes to stimulus selectivity (Miller et al., 2001; Wilent and Contreras, 2005). An interesting example of consistency in sensory input processing across phylogenetically distant species is provided by studies on the short-tailed opossum, which show striking similarities in the pattern of thalamocortical connectivity and in the whisker-evoked synaptic responses of L4 neurons with those observed in mice and rats, despite the absence of barrels in these marsupials (Dooley et al., 2015; Ramamurthy and Krubitzer, 2016).

From L4, sensory signals propagate to the upper layers *via* the prominent axonal projections between L4 spiny neurons and L2/3 pyramidal cells (Feldmeyer et al., 2002; Lefort et al., 2009). L2/3 ITs represent the second level of intracortical processing, distributing information within and beyond their home column through local and long-range corticocortical outputs. Depending on the behavioural context, sensory signals in L2/3 are further modulated by the integration of non-sensory information from other primary and/or associative cortical areas and from the thalamus (Feldmeyer, 2012; Harris and Shepherd, 2015). *In vivo* recordings revealed relatively low spontaneous and evoked firing rates in superficial pyramidal neurons, at least in rodents (de Kock and Sakmann, 2008; Sakata and Harris, 2009; O'Connor et al., 2010; Carton-Leclercq et al., 2023). This sparse firing, likely resulting from a hyperpolarized membrane potential keeping neurons away from their action potential (AP) threshold (Lefort et al., 2009; Carton-Leclercq et al., 2023) and from the recruitment of local inhibitory interneurons (Helmstaedter et al., 2008; Meyer et al., 2011; Haider et al., 2013), suggests that sensory information in superficial layers are encoded through the patterned discharge of fine-scale assemblies of neurons (“sparse coding”) (Harris and Mrsic-Flogel, 2013). Supporting this notion, preferential connectivity between L2/3 cells dedicated to processing of related sensory information has been observed in rodent and cat primary visual cortex (Gilbert and Wiesel, 1989; Yoshimura et al., 2005; Ko et al., 2011), and spatially constrained firing activity has been recorded in response to modality-specific sensory stimulation in the superficial layers of the mouse auditory and somatosensory cortex (Sakata and Harris, 2009; O'Connor et al., 2010).

Deep-layer neurons, which in rat, cat, and primate receive major inputs from L2/3 (reviewed in Thomson and Lamy, 2007), represent the last stage of signal processing within the cortical microcircuit. Engaged in complex computations, these cells combine the results of the successive integrations within the cortical column with converging long-range thalamic and cortical inputs to finally route the output message to specific sets of cortical and subcortical structures. L5 pyramidal neurons, particularly ET cells, exhibit a depolarized membrane potential and rather high spontaneous and sensory-evoked firing frequency *in vivo* (de Kock and Sakmann, 2008; Sakata and Harris, 2009; O'Connor et al., 2010; Carton-Leclercq et al., 2023), consistent with the dense excitatory inputs they receive and their more variable inhibitory innervation (Schubert et al., 2001; Thomson and Lamy, 2007; Petreanu et al., 2009; Feldmeyer, 2012). This suggests that the deep layers of the neocortex may employ a coding strategy based on global variations in firing rates ('dense coding') rather than on the implementation of discrete spatio-temporal dynamics of activity as in the superficial layers (Harris and Mrsic-Flogel, 2013).

Unlike L5 ETs, L6 corticothalamic cells fire at low rate *in vivo*, even in response to various sensory stimuli (Sirota et al., 2005; O'Connor et al., 2010; Oberlaender et al., 2012). Through their projection to the thalamus and to L4, where they innervate GABAergic interneurons strongly in cats but more moderately in rats and mice, L6 corticothalamic cells are strategically positioned to modulate thalamocortical inputs (Thomson, 2010; Pichon et al., 2012). This was demonstrated in the mouse primary visual cortex, where optogenetic stimulation of corticothalamic neurons was shown to modulate the spiking level of upper-layer neurons, *via* the activation of intracortical and intrathalamic inhibitory circuits (Olsen et al., 2012). Moreover, by integrating long-range inputs from higher-order cortical areas, these cells are likely involved in the contextual processing of sensory signals (Zhang and Deschênes, 1998; Feldmeyer, 2012; Vélez-Fort et al., 2014).

Overall, these findings suggest that the fundamental principles governing the functioning of cortical circuits are retained in the different eutherian species studied to date. It would be important to extend this research to a larger number of species, possibly from different mammalian radiations, to assess the extent to which these principles can be generalised to all mammals. Sensory processing in neocortical circuits sharing a relatively similar hodology may be differentially modulated during behaviour. In the mouse visual cortex, visually driven sensory responses in L2/3 pyramidal cells are scaled up as mice transitioned to locomotion (Niell and Stryker, 2010; Polack et al., 2013). This state-dependent facilitation of visual responsiveness has also been observed in monkeys during attention, and even in invertebrate species such as fruit flies during walking or flight (reviewed in Maimon, 2011). Conversely, in the mouse auditory cortex, active behaviour diminishes the gain of tone-evoked spiking responses in superficial layers (Zhou et al., 2014). Similar investigation in humans have produced heterogeneous results, with some studies showing a positive effect of exercise or natural walking on sensory gain and peripheral input processing (Bullock et al., 2015; Cao and Handel, 2019), while others report no modulation of visual sensitivity during treadmill walking (Benjamin et al., 2018).

## 5 Electrophysiological properties of pyramidal neurons

Synaptic potentials generated in the dendrites propagate to the soma and the proximal part of the axon, where they trigger APs when the voltage threshold is reached. The transduction of synaptic inputs into AP-encoded outputs depends not only on synaptic function, but also on the intrinsic membrane properties of neurons that shape the amplitude and kinetics of synaptic events and adjust their ability to elicit firing. Previous research in rodents has shown that the morphology of dendritic trees, together with their electrical properties, strongly influences synaptic integration, excitability, and firing patterns of pyramidal neurons (Mainen and Sejnowski, 1996; Yuste and Tank, 1996; Bekkers and Häusser, 2007; van Elburg and van Ooyen, 2010). This raises the question of whether such morpho-functional interactions are applicable to other species or whether the distinctive anatomical attributes of human neurons confer specific electrophysiological properties. Comparative analysis of the electrical properties of pyramidal neurons between species, and even within the same species, based on *in vitro* studies carried out by different laboratories can prove difficult due to the many experimental factors (e.g., the animal age, composition of intracellular and extracellular solutions, recording temperature or degree of damage to the dendritic tree during slices preparation) that vary from one study to another and are known to affect the synaptic and biophysical properties of neuronal membranes (Hardingham and Larkman, 1998; Zhu, 2000; Bekkers and Häusser, 2007). The task becomes even more complex when considering *in vivo* studies, as the background synaptic activity that characterises intact brain preparations is known to have a significant impact on the excitability and firing of cortical neurons (Destexhe et al., 2003; Altwegg-Boussac et al., 2014; Carton-Leclercq et al., 2021). The increase in the number of data sets and the integration of work combining experiments carried out on several species in a single study have nevertheless enabled a number of electrophysiological characteristics to be compared reliably.

### 5.1 Intrinsic membrane properties

#### 5.1.1 L2/3 pyramidal neurons

The intrinsic excitability of a neuron, which reflects its endogenous capacity to generate APs in response to a given input, is determined by its passive membrane properties, largely conferred by its physical structure, and by its array of non-synaptic voltage-gated ion channels, which mediate a variety of active currents.

**Figure 4:**
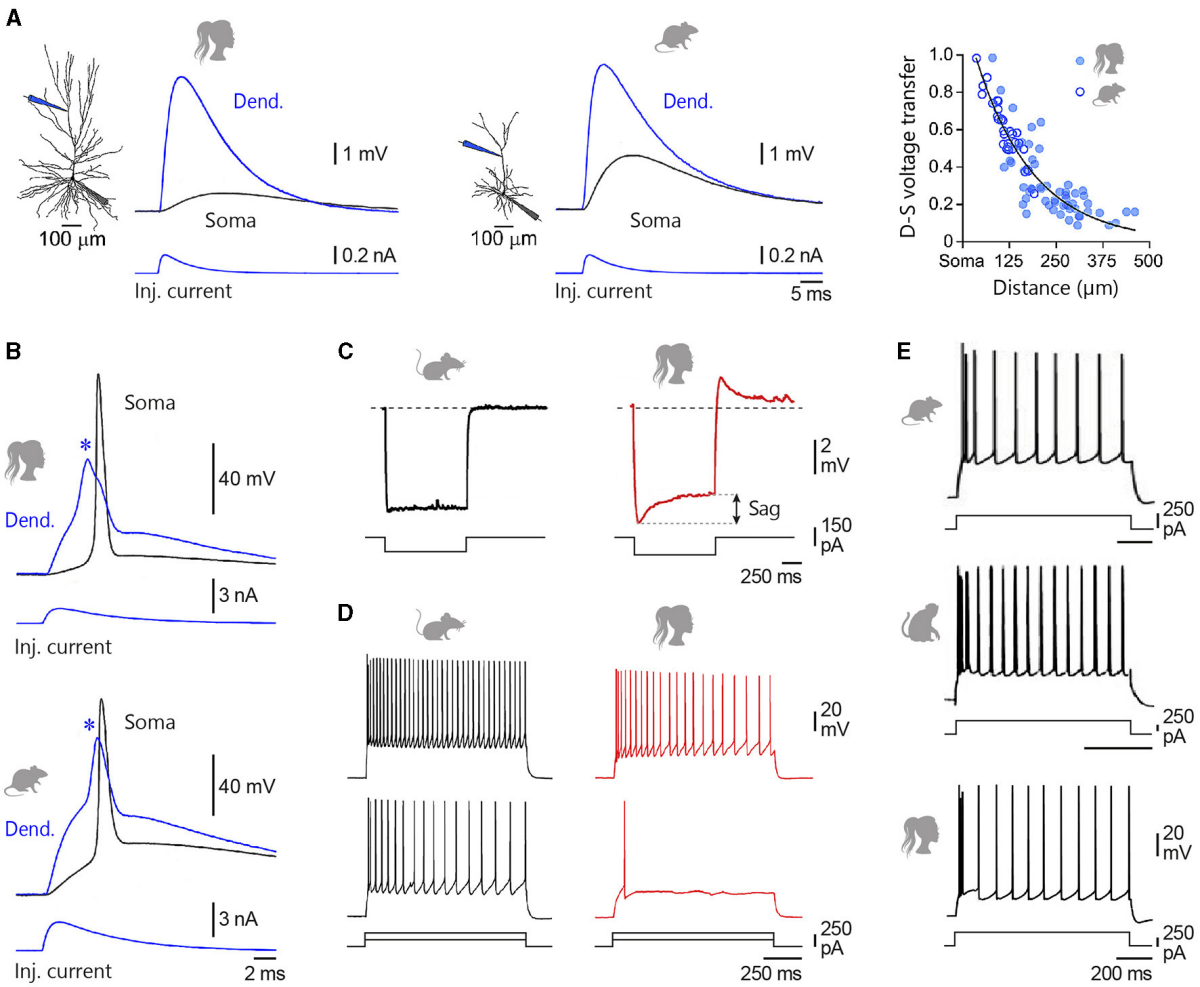
Intrinsic membrane properties of L2/3 pyramidal neurons. **(A)** Simultaneous somatic (Soma, black) and dendritic (Dend., blue) recordings of an EPSP generated at dendritic site by the injection of uniform current waveform (Inj. current, bottom traces) in a human and a rat temporal cortex L2/3 pyramidal neuron. The location of recording electrodes is shown on the reconstructed neurons. The right graph compares the degree of dendro-somatic (D-S) voltage attenuation of simulated EPSPs as they spread from their increasingly remote apical dendritic site of generation to the soma, in human (filled symbols) and rat (open symbols) neurons. **(B)** Evoked dendritic spikes (asterisks) driving somatic AP firing in human and rat L2/3 neurons. **(C)** Voltage responses to hyperpolarizing current steps recorded from a mouse temporal association cortex **(left)** and a human middle temporal gyrus **(right)** L2/3 pyramidal neuron. Double arrow indicates the amplitude of the voltage sag. **(D)** Example voltage responses to depolarizing current steps of +250 and +500 pA for a representative mouse **(left)** and human **(right)** L2/3 pyramidal neuron. **(E)** From top to bottom, Current-evoked firing responses recorded from a rat medial prefrontal cortex, a macaque prefrontal cortex, and a human temporal cortex L2/3 pyramid. Adapted with permission from Gooch et al. (2022) **(A, B)**; Kalmbach et al. (2018) **(C, D)**; van Aerde and Feldmeyer (2015) (rat), González-Burgos et al. (2019) (rhesus monkey), and Deitcher et al. (2017) (human) **(E)**.

According to the cable theory, the passive properties of the dendritic tree are predicted to impose a distortion on synaptic inputs, such that the most distal inputs will yield the most attenuated and prolonged excitatory postsynaptic potentials (EPSPs) at the soma (Rall, 1977; Spruston et al., 1994). Simultaneous somatic and apical dendritic whole-cell recordings from human and rodent L2/3 pyramidal neurons, together with morphologically realistic biophysical modelling, confirmed that somatic EPSP amplitude progressively decreased with increasing distance from the EPSP generation site (Mohan et al., 2015; Eyal et al., 2018; Gooch et al., 2022), and demonstrated that voltage attenuation followed a similar distance-dependence pattern in both species (Figure 4A) (Gooch et al., 2022). The extended and elaborate apical dendritic tree of human pyramidal cells is thus expected to result in strong attenuation of the most distal synaptic inputs. It has been proposed that the cable filtering of human dendrites may be partially compensated for by a lower specific membrane capacitance (~0.5 μF/cm^2^ vs. ~1 μF/cm^2^ in rodent neurons), a biophysical characteristic expected to increase the amplitude and reduce the peak delay of dendritic EPSPs at the soma (Eyal et al., 2016). However, this finding has not been confirmed by recent *in vitro* recordings of L2/3 (Gooch et al., 2022) and L5 (Beaulieu-Laroche et al., 2018) pyramidal cells, suggesting possible heterogeneity in the capacitive membrane properties of human neurons.

In a passive neuronal model, distal EPSPs are expected to exhibit greater somatic temporal summation due to their greater half-width at the soma compared to more proximal EPSPs. However, trains of EPSPs generated at dendritic and somatic sites have been shown to summate similarly in human L2/3 pyramidal neurons (Gooch et al., 2022). This could result from a stronger dendritic expression of hyperpolarization-activated cyclic nucleotide-gated (HCN) channels mediating the h-current (*I*_h_), which is known to reduce the duration of distally generated synaptic events in rat L5 neurons (Williams and Stuart, 2000). Although the spatial distribution of h-channels in L2/3 human neurons remains so far unknown, their presence was evidenced by a pronounced voltage sag (a slow partial repolarization) in response to long hyperpolarizing current pulses delivered at the soma (Deitcher et al., 2017; Kalmbach et al., 2018; Berg et al., 2021; Moradi Chameh et al., 2021), and further supported by *in-situ* hybridisation and single nucleus RNA sequencing studies (Zeng et al., 2012; Kalmbach et al., 2018). Sag ratio values (quantifying the amount of *I*_*h*_) in human supragranular pyramids (11–17%) (Deitcher et al., 2017; Kalmbach et al., 2018) are comparable to those reported in macaques (12–15%) (Zaitsev et al., 2012; González-Burgos et al., 2019), but contrast sharply with their low levels (0.3–2.5%) in homologous neurons in rodents (Figure 4C) (Mason and Larkman, 1990; Larkum et al., 2007; Kalmbach et al., 2018; Berg et al., 2021; but see the relatively large sag ratio reported by van Aerde and Feldmeyer, 2015 in a subpopulation of rat L3 pyramidal cells and by Testa-Silva et al., 2022 in some L2/3 mouse neurons).

The influence of distal dendritic inputs on AP output in human L2/3 neurons is therefore limited by the expansion of their apical dendritic tree and the impact of *I*_h_ on the time course of synaptic potentials. However, recent studies indicate that, similar to rodent neurons (Larkum et al., 1999, 2007; Schiller et al., 2000), human dendrites can initiate local dendritic spikes mediated by voltage-sensitive Na^+^ or Ca^2+^ channels, or dependent on *N*-methyl-d-aspartate (NMDA) receptors, which can increase EPSP amplitude and drive firing at the axon initiation site (Figure 4B) (Gidon et al., 2020; Gooch et al., 2022; Testa-Silva et al., 2022). This suggests that active dendritic electrogenesis could provide a means to compensate for the normalisation of temporal summation and strong voltage attenuation of EPSPs in these neurons. The ability of human neurons to generate NMDA spikes in basal and oblique dendrites was, however, found to be significantly lower than in mouse neurons, probably due to the larger diameter of human dendrites (Testa-Silva et al., 2022). Other work on human neurons has reported briefer apical dendritic Ca^2+^ spikes compared with rodents (Gidon et al., 2020), while a recent study suggested conversely that suprathreshold dendritic integrative operations were conserved between the two species (Gooch et al., 2022). These observations indicate that further research is required to gain a comprehensive picture of how the active properties of human dendrites differ from those of other species.

The ability of EPSPs to initiate firing also depends on the excitability of the somatic membrane. Somatic excitability is generally assessed by measuring the current firing threshold, which is the minimum current of fixed duration required to trigger an AP (an approximation of the rheobase; Lapicque, 1926). This threshold current depends on a combination of membrane parameters, including the resting membrane potential, input resistance (which governs the capacity of a current to modify the membrane potential according to Ohm's law), and the value of the AP voltage threshold. In rodents, removal or occlusion of the dendritic tree increases the intrinsic excitability of individual neurons by increasing input resistance and lowering the AP threshold (Bekkers and Häusser, 2007). Such interactions between dendritic morphology and neuronal excitability seem directly transposable to other species, since pyramidal neurons in the superficial layers of the frontal cortex of the rhesus monkey, which have an elaborate dendritic tree, show reduced somatic excitability compared with their less branched counterparts in the primary visual cortex. Likewise, morphologically related pyramidal neurons, such as those in the visual cortex of mice and monkeys, show similar threshold current values (Amatrudo et al., 2012; Gilman et al., 2017). Finally, a direct comparison of the excitability of human and mouse L2/3 neurons in the same study revealed that human pyramids had a significantly lower input resistance and required the injection of a greater amount of current to be brought to spike (Figure 4D) (Kalmbach et al., 2018; Testa-Silva et al., 2022).

The properties of single somatic APs in L2/3 pyramidal neurons are essentially similar across species: the amplitude (~80–85 mV), voltage threshold (~-40/−50 mV) and half-width duration (~1.0–1.7 ms) measured in humans (Testa-Silva et al., 2014; Deitcher et al., 2017; Moradi Chameh et al., 2021; but see Gooch et al., 2022 for a difference in AP voltage threshold between human and rodent neurons) are well within the ranges reported for monkeys (Amatrudo et al., 2012; Zaitsev et al., 2012; Gilman et al., 2017) and rodents (Lefort et al., 2009; Staiger et al., 2015; Gilman et al., 2017). However, the properties of APs during spike trains may distinguish rodent from human neurons. In particular, human APs have faster onsets than those fired by mouse pyramidal neurons (Testa-Silva et al., 2014). As predicted by theoretical analyses (Ilin et al., 2013), this could enable human neurons to track and encode fast incoming synaptic inputs more efficiently (Testa-Silva et al., 2014; Goriounova et al., 2018). The axon initial segment of human L2/3 pyramids is similarly equipped with a high density of voltage-dependent Na^+^ channels, with a partially segregated distribution of Nav1.2 and Nav1.6 channel subtypes in proximal and distal regions, as observed in rodents (Hu et al., 2009; Tian et al., 2014). Finally, the main firing dynamics identified in human cortical neurons in response to sustained depolarizing current steps (Foehring et al., 1991; Avoli et al., 1994; Deitcher et al., 2017; Kalmbach et al., 2018; Moradi Chameh et al., 2021): regular spike trains with moderate spike frequency adaptation, preceded in some neurons by an initial doublet or triplet of high-frequency spikes, are also commonly observed in the supragranular cortex of monkeys, cats, and rodents (Figure 4E) (rat: Mason and Larkman, 1990; Otsuka and Kawaguchi, 2011; Staiger et al., 2015; van Aerde and Feldmeyer, 2015; cat: Chen et al., 1996; Nowak et al., 2003; monkey: Zaitsev et al., 2012; González-Burgos et al., 2019).

#### 5.1.2 L5 pyramidal neurons

**Figure 5:**
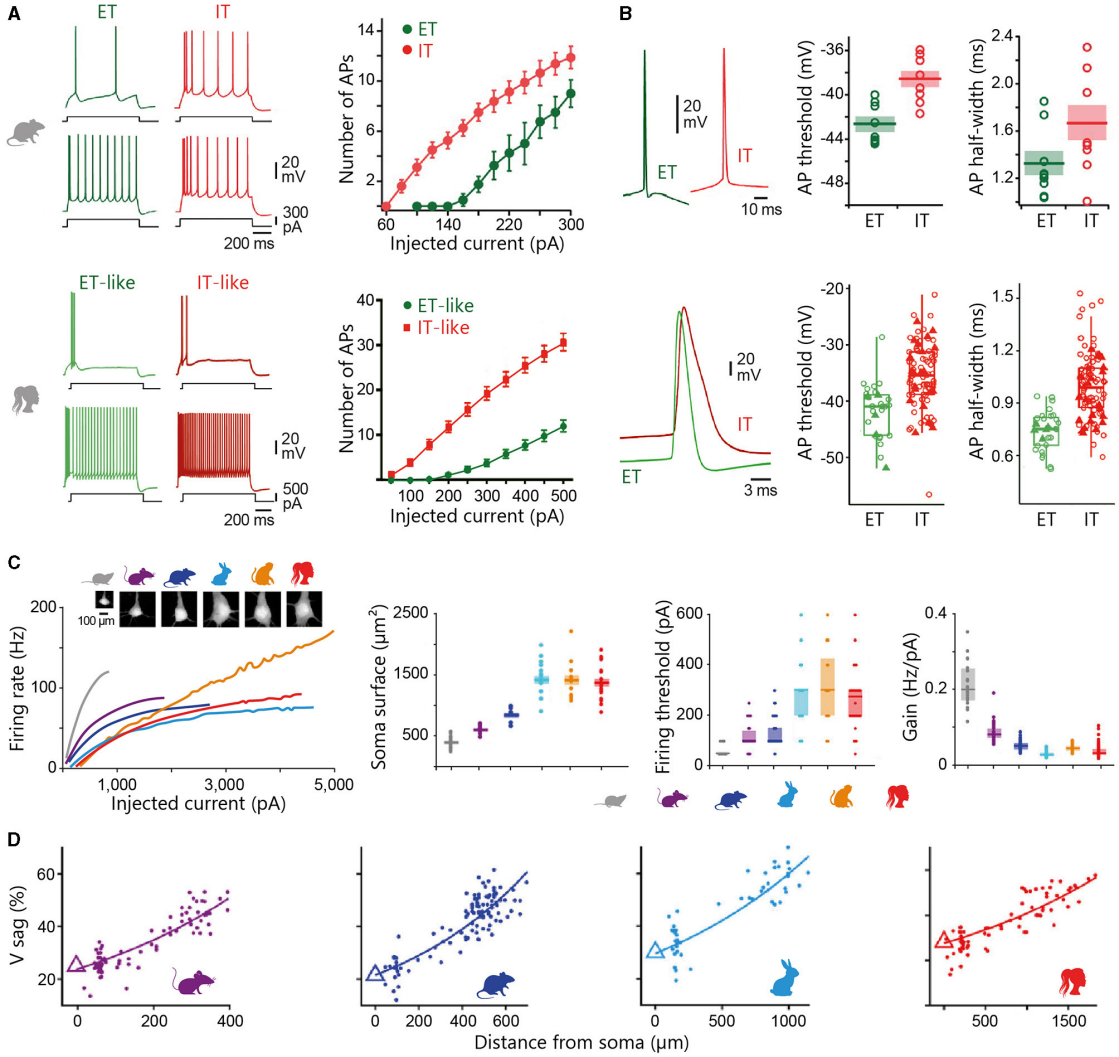
Intrinsic membrane properties of L5 pyramidal neurons. **(A, B)** Electrophysiological properties of infragranular ET and IT neurons in rat medial prefrontal cortex **(top)** and human temporal cortex **(bottom)**. **(A)** Representative voltage responses of ET (green) and IT (red) neurons to depolarizing current steps of increasing intensity **(left)**, and corresponding relationships between injected current intensity and number of evoked spikes **(right)**. **(B)** Example APs in ET and IT neurons and corresponding summary data of their voltage threshold and half-width duration. Data are presented as mean ± s.e.m. in the top graph. Box plots in the bottom graph denote the median and 25–75th percentiles. **(C)** Input-output function of thick-tufted L5 neurons across placental mammalian species. Left, Firing frequency as function of injected current intensity in Etruscan shrew, mouse, rat, rabbit, macaque, and human neurons. Lines illustrate population medians. Two-photon images of example somas are shown on top. Right, Population data of somatic surface area (mean ± s.e.m.), current firing threshold (median and 25–75th percentiles), and input-output neuronal gain (median and 25–75th percentiles). **(D)** h-channel-related membrane properties of L5 thick-tufted neurons. The graphs illustrate the sag ratio as a function of distance from the soma in mouse, rat, rabbit, and human neurons. Triangles represent somatic medians and lines are exponential fits. **(A, B)** are adapted with permission from Dembrow et al. (2010) (rat) and Kalmbach et al. (2021) (human), and **(C, D)** are adapted with permission from Beaulieu-Laroche et al. (2021).

Important differences in subthreshold and suprathreshold intrinsic membrane properties distinguish ET from IT neurons in rodents (Figures 5A, B). Across areas, ET neurons typically exhibit a slightly more depolarized resting membrane potential, lower input resistance, faster membrane time constant and higher threshold current, and frequently (but not always) display reduced input-output gain (assessed by the slope of the relationship between injected current intensity and firing frequency) (Figure 5A) (Mason and Larkman, 1990; Hattox and Nelson, 2007; Dembrow et al., 2010; Oswald et al., 2013; Suter et al., 2013; Rock and Apicella, 2015; Anastasiades et al., 2018; Baker et al., 2018b; Kalmbach et al., 2021; but see Groh et al., 2010 for regional variations in biophysical parameters differentiating IT from ET neurons). These properties are largely conserved in human and non-human primates, where ET-like cells are found to be less excitable than their slender neighbours, both at rest and during integration of inputs of varying intensity (Figure 5A) (Beaulieu-Laroche et al., 2018, 2021; Bakken et al., 2021; Kalmbach et al., 2021; Moradi Chameh et al., 2021). Across species, the excitability of L5 ETs generally correlates negatively with their size, such that thick-tufted pyramidal neurons of the Etruscan shrew are more easily driven to fire APs than their counterparts in rodents, macaques and humans (Beaulieu-Laroche et al., 2018, 2021; Bakken et al., 2021; Kalmbach et al., 2021) (Figure 5C). However, variations between ET and IT projection subtypes in a given species often appear to be greater than differences between species, at least for temporal cortex neurons (Kalmbach et al., 2021). Consistent with findings in other placental mammals, ET cells in humans have a more prominent voltage sag (~25–30% sag ratio) than IT neurons (~15%), associated with a progressive increase in h-channel density with increasing distance from the soma (Figure 5D) (Kole et al., 2006; Beaulieu-Laroche et al., 2018, 2021; Kalmbach et al., 2021; Moradi Chameh et al., 2021). The differences in the amount of *I*_*h*_ between the two subtypes of L5 neurons are expected to contribute to their specific passive membrane properties and to shape their subthreshold integrative properties, like in rodents where h-channels impart ET neurons with a less pronounced temporal summation of EPSPs than in IT cells (Dembrow et al., 2010; Sheets et al., 2011; Kalmbach et al., 2015; Anastasiades et al., 2018). When activated by sustained depolarization, primate and rodent L5 ET neurons show less frequency adaptation (progressive increase in the inter-spike interval during a train of APs) than IT cells (Figure 5A). Individual APs in ETs also exhibit faster kinetics, shorter duration and a lower voltage threshold (Figures 5A, B) (rodent and primate: Bakken et al., 2021; Beaulieu-Laroche et al., 2021; Kalmbach et al., 2021; rodent: Mason and Larkman, 1990; Hattox and Nelson, 2007; Dembrow et al., 2010; Oswald et al., 2013; Suter et al., 2013; Pathak et al., 2016). The thinner APs of L5 corticofugal neurons could limit calcium influx and activation of calcium-dependent potassium channels responsible for spike frequency adaptation during AP trains (Faber and Sah, 2003), and thus contribute to their more regular firing pattern (Suter et al., 2013).

Compared to L5, the intrinsic parameters that discriminate ET and IT populations in L6 are more difficult to identify. While some studies describe L6 ET neurons as having faster time constants, shorter APs and higher rheobase than IT cells as in L5 (Kumar and Ohana, 2008; Crandall et al., 2017), others report lower threshold currents, longer APs (Kinnischtzke et al., 2016), as well as higher input resistance values (Kinnischtzke et al., 2016; Crandall et al., 2017; Yang et al., 2022). However, weaker steady-state adaptation ratios and relatively larger voltage sags are biophysical properties that also consistently separate ETs from ITs in L6 (Kumar and Ohana, 2008; Vélez-Fort et al., 2014; Crandall et al., 2017; Yang et al., 2022; but see Cotel et al., 2018).

A significant proportion (~10–30%) of L5 ET neurons in cats and rats can fire in bursts (clusters of APs with short inter-spike intervals riding on a depolarizing envelope) in response to threshold current injections (rodent: Chagnac-Amitai et al., 1990; Mason and Larkman, 1990; Dégenètais et al., 2002; Paz et al., 2009; Groh et al., 2010; cat: Baranyi et al., 1993; Nuñez et al., 1993; Nowak et al., 2003). This firing behaviour is nonetheless not unique to ETs as burst firing can be observed *in vivo* and *in vitro* in L5 corticostriatal neurons in rodents (Yang et al., 1996; Pidoux et al., 2011b), and to a lesser extent, in the upper layers of sensorimotor and visual cortices in cats (Nishimura et al., 2001; Nowak et al., 2003). The traditional classification of L5 pyramidal cells into regular spiking or intrinsically bursting subtypes is further complicated by the fact that firing dynamics evolve *in vivo* with the level of background synaptic activity, which is function of vigilance state and behaviour (Mahon et al., 2001; Steriade, 2004; Altwegg-Boussac et al., 2014). Across species, *in vivo* recordings generally describe L5 ETs as more spontaneously active than ITs and more prone to generate spike bursts (monkey: Pasquereau and Turner, 2011; rat: Ushimaru and Kawaguchi, 2015; Rojas-Piloni et al., 2017; Saiki et al., 2018), in stark contrast to the sparse spiking activity of L6 corticothalamic neurons (rabbit: Swadlow, 1989; cat: Sirota et al., 2005; rat: Oberlaender et al., 2012). Burst firing is suggested to be less frequent in human and non-human primates than in rodents and carnivores (Avoli and Olivier, 1989; Foehring et al., 1991; Chang and Luebke, 2007; Beaulieu-Laroche et al., 2018, 2021; Moradi Chameh et al., 2021; Piette et al., 2021). This reduced bursting capacity of human neurons could result from an increased electrical isolation of distal dendrites due to their length (Beaulieu-Laroche et al., 2018, 2021), which is expected to limit electrical coupling between the different neuronal compartments and, thus, the influence of dendritic spikes on somatic output (Larkum et al., 1999, 2001; Williams and Stuart, 1999). However, such a decrease in calcium dendritic electrogenesis in humans was not reported by another study (Kalmbach et al., 2021), suggesting variability in the ability of human dendrites to trigger regenerative potentials, as in rodents (Helmchen et al., 1999; Fletcher and Williams, 2019). As postsynaptic bursts are thought to influence synaptic integration, plasticity, and information signalling in cortical circuits (Lisman, 1997; Larkum et al., 1999; Williams and Stuart, 1999), the lower intrinsic burst capacity of human neurons could appear as disadvantageous to cortical computations. Alternatively, as suggested by theoretical studies, electrically isolated individual dendrites could function as separate processing units, increasing the possibilities for independent parallel operations and thus, the computational capabilities of cortical neurons (Häusser and Mel, 2003; Eyal et al., 2018).

ET and IT pyramidal neurons also differ in their responses to neuromodulatory transmitters (for reviews, see Shepherd, 2013; Dembrow and Johnston, 2014; Radnikow and Feldmeyer, 2018). For instance, cholinergic inputs are more effective at promoting persistent spontaneous firing in deep-layer corticofugal neurons than in corticocortical cells (Dembrow et al., 2010; Joshi et al., 2016; Baker et al., 2018b), an effect likely involving inhibition of M-current mediating K^+^ channels and activation of non-specific cationic conductances (Baker et al., 2018b). Similarly, the increase in cortical excitability induced by activation of alpha2-noradrenergic receptors is significantly reduced in ITs compared with ETs (Dembrow et al., 2010). The responsiveness of pyramidal neurons to dopaminergic and serotoninergic afferents also varies according to their projection targets, owing to the differential expression of neuromodulator receptor subtypes (Avesar and Gulledge, 2012; Gee et al., 2012). Given the critical roles of neuromodulatory systems in regulating sleep, wakefulness, motor control, and various high cognitive functions such as conscious perception, attention, learning, and memory, a comprehensive knowledge of the neuromodulation of projection neuron subpopulations in humans will be essential for a cell-type-specific mechanistic understanding of numerous brain functions and dysfunctions, as well as for the development of potential treatments (Shepherd, 2013; Ma et al., 2018).

### 5.2 Excitatory synaptic function

Recent cutting-edge studies, combining whole-cell recordings of pairs of L2/3 pyramidal neurons with the identification of putative synaptic contacts, have begun to characterise excitatory synaptic transmission in the supragranular layers of the human neocortex. Despite potential differences in the capacity to form synaptic connexions, given the higher density of spines on human neurons (Benavides-Piccione et al., 2002), the rate of local functional connectivity (around 12% in mice, 14% in rats, and 14% in humans) and the number of synapses per connexion (between ~2 and 4) are reported to be comparable between rodents and humans. This similarity extends to the median amplitude (~0.5 mV despite substantial differences between studies and cortical regions, see de Kock and Feldmeyer, 2023 for review), latency, and kinetics of unitary (evoked by the firing of single presynaptic neurons) EPSPs reaching the soma, which show remarkable consistency across a variety of species (human and mouse: Testa-Silva et al., 2014; Szegedi et al., 2016; Seeman et al., 2018; Campagnola et al., 2022; Hunt et al., 2023; mouse: Oswald and Reyes, 2008; Lefort et al., 2009; Ko et al., 2013; Jouhanneau et al., 2015; Luo et al., 2017; rat: Mason et al., 1991; Hardingham and Larkman, 1998; Holmgren et al., 2003; Yoshimura et al., 2005; Feldmeyer et al., 2006; Hardingham et al., 2010; macaque and rat: Povysheva et al., 2006; cat and rat: Thomson et al., 2002). It also predicted that a similarly small number of simultaneously active L2/3-L2/3 synapses (between ~125 and 145) is required to generate a somatic AP in human and rat cell models (Eyal et al., 2018). This maintenance of the fundamental properties of excitatory synaptic transmission between L2/3 human neurons, despite a strong voltage attenuation of dendritic EPSPs and lower somatic input resistance, could result from compensatory mechanisms, such as initiation of dendritic spikes (see above) and/or an increase in AMPA and NMDA synaptic conductances, as predicted by modelling studies (Eyal et al., 2018; Hunt et al., 2023). L2/3 pyramidal-to-pyramidal synaptic connexions in humans are, however, considered to be more reliable than in rodents, with a failure rate between functionally connected cells ranging from 0 to 8% compared with 3.2 to 25% in mice and rats (human: Szegedi et al., 2016; human and mouse: Hunt et al., 2023; mouse: Oswald and Reyes, 2008; Jouhanneau et al., 2015; rat: Hardingham and Larkman, 1998; Koester and Johnston, 2005; Feldmeyer et al., 2006; Hardingham et al., 2010).

Connexions between pyramidal cells and GABAergic interneurons generally produce larger synaptic responses than connexions between pyramidal cells, partly because of the higher input resistance of interneurons (rat and cat: Thomson et al., 2002; rat and macaque: Povysheva et al., 2006; rat: Holmgren et al., 2003; human: Molnár et al., 2008). The amplitude distribution of unitary EPSPs at this synapse often shows a long tail formed by large amplitude synaptic events, although this can also be observed at other types of connexions (Holmgren et al., 2003; Lefort et al., 2009). Interestingly, dual whole-cell patch clamp recordings in the human neocortex have identified a subset of supragranular pyramidal cells establishing particularly strong synapses on fast-spiking interneurons and producing excitatory events large enough to drive postsynaptic firing and initiate a complex sequence of polysynaptic activity in the local network (Molnár et al., 2008; Szegedi et al., 2016). The properties of human inhibitory synapses and their impact on local circuit function are still not fully established. Nevertheless, the consistency of long-lasting inhibitory responses elicited by neurogliaform interneurons in rodent and human L2/3 pyramidal neurons (Tamás et al., 2003; Oláh et al., 2007), along with the comparable facilitatory effect of acetylcholine on Martinotti cell-mediated lateral inhibition in both species (Obermayer et al., 2018), suggest that these interneuron subtypes may similarly modulate synaptic input processing at distal dendrites in humans.

Synaptic function in human L5 is just beginning to be uncovered. The estimated rate of functional connectivity between L5 pyramidal neurons is similarly low (~10%) in humans and rodents (rat: Markram et al., 1997; Lefort et al., 2009; human: Seeman et al., 2018; Campagnola et al., 2022). Quantitative analysis of the structural correlates of synaptic transmission indicates that most L5 synaptic boutons in human temporal cortex present a single active zone comparable in size to those in L4 and L5B of the rat somatosensory cortex, despite substantial individual variability. The number of synaptic vesicles in the putative readily releasable pool of L5 neurons is similar in both species, but the resting pool is at least 2-fold larger in humans, which could facilitate rapid refilling of the releasable pool during sustained high-frequency activity (human: Yakoubi et al., 2019; rat: Rollenhagen et al., 2015, 2018). Finally, it is suggested that the mean amplitude of the unitary EPSP at the L5 pyramidal-to-pyramidal connexion (including ET and IT neurons) is comparable between species (~0.6 mV), but further human studies are needed to confirm these results (rodent: de Kock and Feldmeyer, 2023 for review; see also Lefort et al., 2009; Hardingham et al., 2010; Kerr et al., 2013; human: Seeman et al., 2018).

#### 5.2.1 Plasticity of synaptic transmission

Connexions between L2/3 pyramidal cells generally exhibit frequency-dependent short-term synaptic depression *in vitro*. This rapid decrease in the amplitude of the postsynaptic response during trains of presynaptic spikes, primarily mediated by presynaptic mechanisms involving a progressive inactivation of Ca^2+^ channels in synaptic terminals and reduction of the pool of readily releasable synaptic vesicles (Zucker and Regehr, 2002; Nanou and Catterall, 2018), can be similarly observed in humans, cats, and rodents (mouse and human: Testa-Silva et al., 2014; Campagnola et al., 2022; Hunt et al., 2023; mouse: Oswald and Reyes, 2008; Luo et al., 2017; but see Jouhanneau et al., 2015 for an overall stability of excitatory synaptic responses *in vivo*; rat: Holmgren et al., 2003; Koester and Johnston, 2005; Feldmeyer et al., 2006; cat: reviewed in Thomson and Lamy, 2007). However, human L2/3 excitatory connexions have been shown to recover faster from short-term depression than synapses in rodents, a property that is expected to increase information transfer within the cortical circuit (Testa-Silva et al., 2014; Campagnola et al., 2022; Hunt et al., 2023).

The timing-based learning rules governing the sign of long-term synaptic plasticity in L2/3 pyramidal cells diverge between humans and rodents. In juvenile rodents, pairing protocols in which an extracellularly-evoked EPSP precedes the current-evoked postsynaptic firing by a short time interval (pre-post intervals between 0 and +20 ms) typically induce long-term potentiation (LTP), whereas long-term depression (LTD) is triggered when the postsynaptic activity precedes the presynaptic stimulation for a broader range of time differences (pre-post intervals between 0 and −40 ms) (Feldman, 2000; Froemke and Dan, 2002). In contrast, LTP at adult human synapses is induced with a wider temporal window extending approximately from −100 to +5 ms, and LTD for pre-post intervals between +10 and +20 ms (Verhoog et al., 2013). Differences in developmental stage (i.e., juvenile vs. adult) may partly explain this disparity, as the ability of excitatory synaptic connexions to produce LTD has been shown to decrease with age in rodents (Banerjee et al., 2009; Verhoog et al., 2013). Beyond these differences in timing rules, the layer-specific cholinergic regulation of long-term synaptic plasticity in the rodent neocortex seems to be retained in humans (Obermayer et al., 2017 for review). Importantly, the variable firing patterns of cortical neurons *in vivo* (Shadlen and Newsome, 1998; O'Connor et al., 2010) are expected to give rise to multiple and complex spike-timing interactions between pre- and post-synaptic neurons, further complicating the rules for plasticity induction (Sjöström et al., 2001).

## 6 Temporal organisation of cortical activity

In an intact brain, individual neurons defined by their specific intrinsic membrane properties interact synaptically to give rise to the ongoing *in vivo* dynamics of cortical circuits (Llinás, 1988). Depending on the behavioural context, mammalian neurons can form cell assemblies of different sizes capable of generating oscillatory activities at various frequencies (Steriade, 2000; Buzsáki and Draguhn, 2004). These self-organised patterns of activity play a crucial role in determining the global functional state of the brain and contribute to a range of executive and cognitive functions, including selection of input signals, sensorimotor integration, regulation of information transfer across distributed networks, motor action, and contextual formation and storage of subjective percepts (Engel et al., 2001; Salenius and Hari, 2003; Buzsáki and Draguhn, 2004; Fries, 2015).

By carefully placing electroencephalographic (EEG) electrodes on the skulls of rabbits and dogs, Caton (1875) was the first to discover, in 1875, that a living brain constantly generates fluctuating electrical activity, even in the absence of sensory stimulation or motor activity. A few decades later, the German psychiatrist Berger (1929), secretly conducting human EEG recordings at night in the basement of his university, understood that this ongoing cerebral activity was temporally structured in the form of oscillations whose amplitude and frequency depended on the behavioural or mental state, and introduced the idea that a given cerebral state could be defined by a specific profile of EEG activity. Since the seminal works of Caton and Berger, EEG recordings have been widely used in a variety of mammals to characterise brain dynamics during the sleep-wake cycle and their disruption in neurological disorders (Uhlhaas and Singer, 2006).

### 6.1 Physiological rhythms of sleep and wakefulness

#### 6.1.1 Slow-wave sleep

All mammals examined so far exhibit some form of sleep, whose mean daily duration varies from 2 h in the African bush elephant to 20 h in the little brown bat (Siegel, 2022). High-amplitude, low-frequency (0.5–4.0 Hz) cortical oscillations are the most prominent pattern of electrical activity during the deep phases of slow-wave sleep (SWS), a sleep state generally characterised by behavioural quiescence, raised arousal threshold, reduced but steady electromyographic, cardiac and respiratory activities, and rapid reversibility to wakefulness (Blake and Gerard, 1937; Jouvet, 1967).

**Figure 6:**
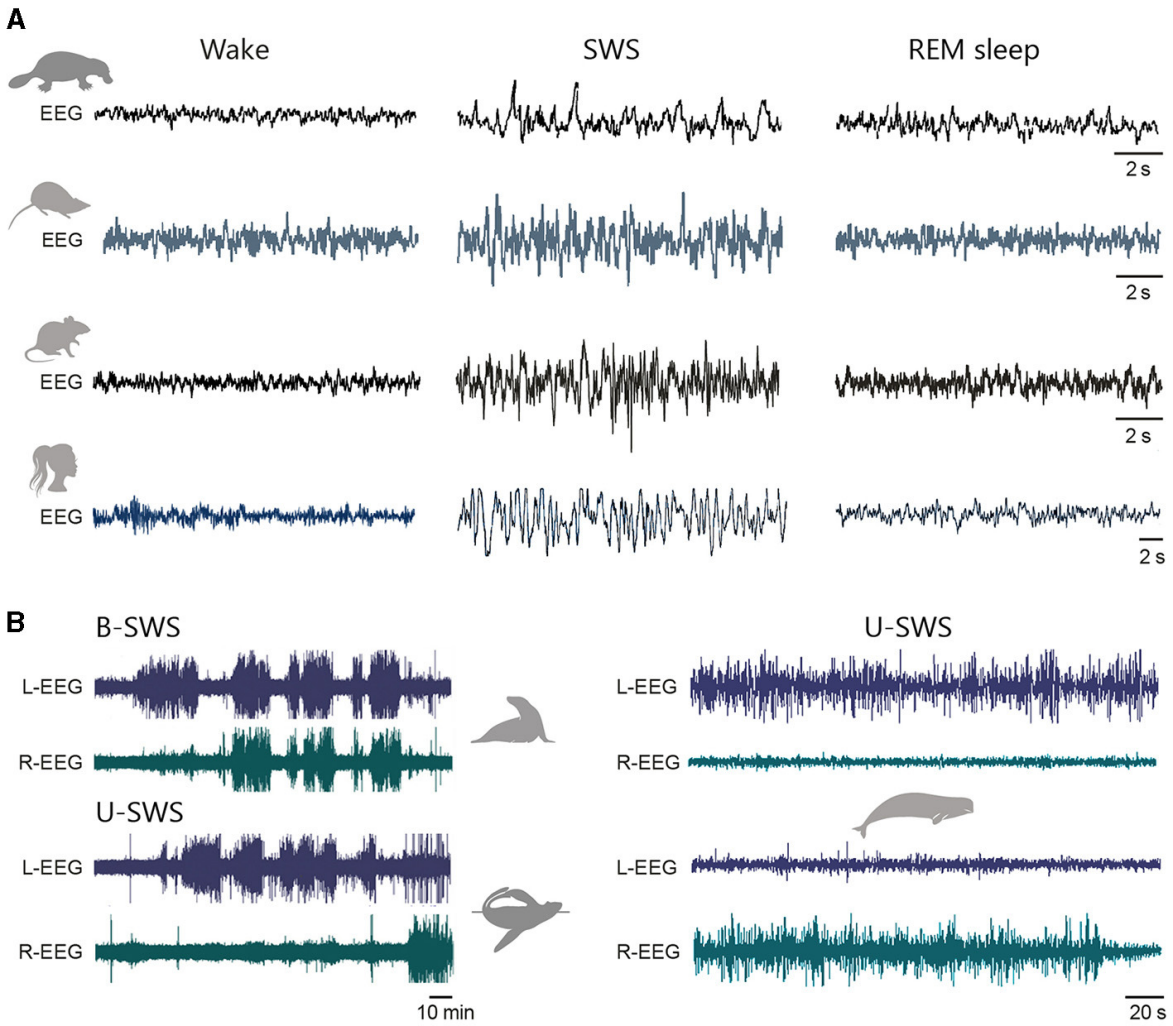
Conservation of sleep-wake EEG patterns across mammals. **(A)** Example traces of EEG activity recorded from a monotreme (platypus), a marsupial (dusky antechinus), and two placental (rat and human) species during active wakefulness (wake), deep stages of slow-wave sleep (SWS), and rapid eye movement sleep (REM). Note the similarity of morphology and frequency of EEG waveforms across species. A moderate-voltage EEG recording is illustrated for platypus REM sleep (see text). For each species, the EEGs in the three states of alertness are presented on the same scale. Data are adapted from Siegel et al. (1999) (platypus); Zaid et al. (2022) (dusky antechinus); Mahon et al. (2006) (rat); Stickgold and Walker (2010), Liu and Dan (2019) (human). **(B)** Bilateral EEG recordings (L: left, R: right) from a semi-aquatic northern fur seal during SWS, showing bilateral high-voltage slow waves when animals are sleeping on land (BSWS, top) and unihemispehric slow-wave activity when sleeping in the sea (USWS, bottom). Belugas spend most of their sleeping time in a unihemsipheric SWS **(right)**. Adapted with permission from Lyamin et al. (2018) (fur seal) and Lyamin et al. (2008) (beluga).

Extracellular and intracellular recordings in cats and rodents have shown that EEG slow waves result from the rhythmic alternation between prolonged periods of membrane depolarization and firing, and episodes of silence and hyperpolarization in large ensembles of cortical neurons (Steriade et al., 1993a; Sanchez-Vives and McCormick, 2000; Mahon et al., 2001, 2006; Chauvette et al., 2011). This slow cortical rhythm and associated behavioural correlates are largely conserved in placental mammals (Figure 6A). However, while slow oscillations typically occur bilaterally in terrestrial mammals, cetaceans (whales and dolphins) spend 70–90% of their sleep time in unihemispheric SWS; a unique cortical state where one cerebral hemisphere exhibits slow-wave EEG, while the other shows wake-like EEG activity (Figure 6B) (Lesku et al., 2006; Lyamin et al., 2008). During unihemispheric SWS, the eye contralateral to the “asleep” hemisphere is usually closed and the eye contralateral to the “awake” hemisphere remains open (Lyamin et al., 2004). Cetaceans engaged in unihemispheric SWS can rest on the bottom, float at the surface or swim slowly in a single direction, with periodic fin movements aiding in posture stabilisation. It has been suggested that the unusual phenomenology of cetacean sleep may serve to maintain a certain level of motor activity, enabling them to come to the surface to breathe, to remain alert to potential threats, or for thermogenesis (Lyamin et al., 2008). Semi-aquatic fur seals represent a remarkable variant of this adaptation to environmental conditions, as they display large amounts of bilateral high-voltage slow activity when sleeping on land and large amounts of interhemispheric EEG asymmetry when sleeping in water (Figure 6B) (Lyamin et al., 2018). The neural mechanisms behind the lateralized nature of SWS in cetaceans remain a mystery. While the reduced interhemispheric synchronisation of SW activity in mammals with callosotomy or callosal agenesis (Singer and Creutzfeldt, 1969; Nielsen et al., 1994; Mohajerani et al., 2010) suggests that the relatively small corpus callosum of cetaceans may be involved, such callosal reduction has not been found in fur seals (Lyamin et al., 2008).

Polygraphic correlates of SWS in marsupials closely resemble those of terrestrial placental mammals (Van Twyver and Allison, 1970; Zaid et al., 2022), despite variation in total sleep duration or SWS proportion between species (Siegel, 2005). Monotremes also show similar periods of quiet sleep with high-voltage EEG and reduced motor activity, for 6 to 8 h per day (Figure 6A) (Siegel et al., 1999; Siegel, 2022).

#### 6.1.2 Paradoxical sleep

Episodes of rapid-eye-movement (REM) or paradoxical sleep usually follow periods of SWS. In marsupials and placentals, the slow, and globally synchronised EEG waves of the SWS are here replaced by rapid, low-voltage oscillations in the beta/gamma range (typically at 20–40 Hz) (Figure 6A) (Llinás and Ribary, 1993; Steriade et al., 1996, 2001; Zaid et al., 2022). These high-frequency cortical activities are accompanied by a regular hippocampal theta rhythm observable in the overlying neocortex, with variations in the duration and properties of theta periods across species (Robinson et al., 1977; Cantero et al., 2003). This sleep state is particularly intriguing because it combines reduced perceptiveness of the external world and almost complete muscular atonia with a high level of cerebral activity resembling that of wakefulness, irregular cardiac and respiratory rhythms and frequent phasic movements of the eyes and extremities (ears, vibrissae, fingers, beak, tail) (Aserinsky and Kleitman, 1953; Dement, 1958; Jouvet, 1967). Remarkably, this is also the period when our dreams are most vivid and take on a fantastic character. The sparse and often inconsistent reports of mammalian-like REM sleep in fish, amphibians, and reptiles suggests that this sleep state may have appeared with endothermy in birds and mammals (Lesku et al., 2006; Hartse, 2011). Sometimes described as a state of “heightened inward arousal,” one of the proposed functions of REM sleep could be to prepare the brain to wake up in a more alert state, thereby avoiding the poor sensory-motor function and high vulnerability associated with waking up during SWS, which would be detrimental for survival (Snyder, 1966).

Monotremes appear to have an unusual form of REM sleep (Siegel et al., 1998). In platypuses, sleep phases characterised by rapid eye movements and intermittent twitching of the beak and head are associated with moderate- or high-voltage EEG waves (Figure 6A) (Siegel et al., 1999). Such an EEG profile, associated with an irregular firing of brainstem neurons typical of REM sleep in placentals and marsupials, has also been observed in the short-beaked echidna (Siegel et al., 1996, 1998). However, these findings need to be confirmed, since another study conducted in the echidna has reported periods of REM sleep with EEG activities close to those described in therian mammals (Nicol et al., 2000). The daily duration of the REM sleep-like state in monotremes (~7.5 h/day; the platypus is reputed to have more REM sleep than any other mammal) exceeds that of marsupials (~4.4 h/day) and placentals (~2 h/day) (Siegel, 2022). Given that monotremes have a lower body and brain temperature (~31°C) as compared to other mammals (~35.5°C in marsupials and 37°C in placentals), it has been hypothesised that prolonged periods of REM sleep, which are associated with a warming of various brain regions (Wehr, 1992), may contribute to the maintenance of thermal conditions compatible with autonomic function and alert awakening (Siegel, 2022). It has also been suggested that the EEG characteristics of therian SWS and REM sleep may have evolved from a single, phylogenetically older sleep stage combining features of both states, and that cortical activation (fast and low-voltage cortical activity) during REM sleep may be a more recent acquisition (Siegel et al., 1998; Siegel, 2022).

To date, there is no unequivocal proof of the existence of REM sleep in cetaceans. Behavioural signs typical of REM sleep in terrestrial mammals (muscle twitches, erections in males, eyelid movements) have been documented in several cetacean species but do not appear to be specific to this sleep state. However, it is possible that REM sleep episodes in cetaceans are too short to be reliably recorded, or that REM sleep is present in these species in a modified form (Lyamin et al., 2008). Again, it is interesting to note that the average daily amount of REM sleep in fur seals is much lower when they sleep in the water than when they sleep on land (Lyamin et al., 2018).

#### 6.1.3 Wakefulness

Cortical activity during wakefulness continuously varies according to the level of attention, the behavioural context or overall motor activity. As first demonstrated by Hans Berger and Edgar Adrian, alpha-frequency oscillations (7–12 Hz) appear on the EEG during quiet wakefulness (especially when eyes are closed) and predominate in posterior brain regions in primates and carnivores (Berger, 1929; Adrian and Matthews, 1934; Lopes da Silva et al., 1973; Bollimunta et al., 2008). Not all human beings are equally endowed with an occipital alpha rhythm and a small proportion of individuals even show an EEG almost entirely devoid of alpha waves after childhood (Niedermeyer, 1997). A second form of alpha oscillations, occurring at 7–20 Hz (also known as alpha-type mu rhythm), is observed in the frontal and parietal regions of rodents and cats, as well as in the central regions of humans, during periods of motor idleness preceding the completion of a sensory-motor task (Gastaut, 1952; Rougeul et al., 1972; Buzsaki et al., 1988; Pfurtscheller et al., 1996).

The transition from quiet to active wakefulness (i.e., associated with motor activity, attentional or behavioural processes) is manifested by a clear change in the EEG pattern in most cortical areas. This is characterised by a decrease in the proportion of cortical waves below 10 Hz, replaced by activities in the beta and gamma frequency bands (mainly in the 20–60 Hz range). These fast rhythms reflect changes in the ongoing subthreshold synaptic activity of cortical pyramidal neurons, which undergo a slight depolarization and shift from relatively slow and large oscillations to fast and small-amplitude synaptic potentials. The impact of membrane depolarization on cell firing varies by layer and cortical region, with pyramidal neurons in deeper layers showing a more pronounced increase in firing rate (reviewed in Poulet and Crochet, 2019). High-frequency activities are a hallmark of active waking in placentals (Steriade, 2003; Buzsáki et al., 2013), marsupials (Van Twyver and Allison, 1970; Zaid et al., 2022), and monotremes (Figure 6A) (Siegel et al., 1996, 1999), although the latter two groups have been less extensively studied. Cortical activity during waking is regulated by several groups of neurons that release neuromodulatory substances, including acetylcholine, noradrenaline, histamine, and serotonin, among others. These neurons are active during wakefulness, while their firing is reduced or suppressed during SWS (Lin, 2000; Jones, 2005). Notably, ascending cholinergic inputs from the basal forebrain and pontine tegmental nuclei play an prominent role in promoting and maintaining arousal through direct effects on the excitability and activity of cortical cells, and indirectly, by activating thalamocortical glutamatergic neurons (Metherate et al., 1992; Steriade et al., 1993a,b; Pinto et al., 2013; Eggermann et al., 2014). High-frequency oscillations during sensory processing arise from small neural networks of interconnected inhibitory and pyramidal cells (Buzsáki and Wang, 2012), but locally generated gamma activities can transiently synchronise over long distances during periods of heightened attention or conscious perception in primates (Melloni et al., 2007; Gregoriou et al., 2009). One well-documented mechanism for preserving the frequency of physiological rhythms and their inter-areal coherence in brains of different sizes is the scaling of the diameter of a fraction of myelinated axons. This scaling is expected to accelerate the propagation of APs in large brains, ensuring that homologous brain regions in different species can communicate within roughly the same time frame (reviewed in Buzsáki et al., 2013).

### 6.2 Pathological oscillations in epileptic and dying brains

If coordinated spatio-temporal activity patterns are the basis of normal brain functioning, their disruption is expected to lead to functional abnormalities. Indeed, Parkinson's disease, schizophrenia, epilepsy and certain forms of coma are all examples of pathologies whose clinical symptoms are associated with disturbances in cortical oscillatory activities (reviewed in Uhlhaas and Singer, 2006). Although there is substantial literature on a variety of brain disorders, I will focus here on two examples of abnormal neural synchronisation: spike-wave discharges in absence epilepsy and the altered cortical patterns observed in the dying brain.

#### 6.2.1 Absence epilepsy

Childhood absence epilepsy, an epileptic syndrome characterised by recurrent generalised non-convulsive seizures, provides an interesting example of disturbed brain dynamics identifiable in several mammals, with fairly similar behavioural consequences. Typical absence seizures involve brief episodes of impaired consciousness, manifested by a sudden interruption of voluntary sensory-motor behaviour, staring, unresponsiveness and retrograde amnesia of the epileptic episode. Secondary clinical symptoms, such as automatisms or mild clonic movements of the eyes, eyelids, mouth or limbs, are also commonly observed. Occurring frequently, up to hundreds of times a day, these seizures can interfere with the normal cognitive and psychosocial development of children (Blumenfeld, 2005a; Cavanna and Monaco, 2009; Crunelli et al., 2020). Absence seizures result from abnormally synchronised oscillations in corticothalamocortical networks, evident as high-amplitude spike-waves at 3–4 Hz on human EEG (Depaulis and Charpier, 2018; Crunelli et al., 2020). Numerous investigations of absence seizure patient populations using high-density EEG recordings, MEG and functional MRI have demonstrated that epileptic oscillations are initiated in a circumscribed region of the neocortex before their generalisation (Holmes et al., 2004; Westmijse et al., 2009; Bai et al., 2010). The severity of clinical symptoms has been shown to correlate with the duration, intensity and inter-regional coherence of spike-wave discharges (Blumenfeld, 2005b; Guo et al., 2016).

Spike-waves are observed not only in humans, but also in monkeys (Steriade, 1974), cats (Gloor and Fariello, 1988), and ferrets (Youngblood et al., 2015), where they have a similar frequency. The cortically-initiated spike-waves in rodents exhibit a relatively faster frequency of 5–10 Hz, despite similar behavioural correlates (altered sensory responsiveness, behavioural arrest, vibrissae or jaw muscle twitching, occasional chewing) and pharmacological sensitivity to anti-absence medications (Polack et al., 2007, 2009; Depaulis et al., 2016; Jarre et al., 2017). The reason for the higher frequency of spike-wave oscillations in rodents is not yet well understood, but experimental and theoretical work has proposed that it may involve species differences in the prevalence of GABAergic receptor subtypes affecting the duration of the oscillatory cycle (Destexhe, 1999; Destexhe et al., 1999; Sanchez-Vives et al., 2021). Consistent findings in cats and rats show that these abnormal oscillations dynamically alter the intrinsic excitability of cortical neurons, impeding their ability to integrate and process sensory information efficiently (Neckelmann et al., 2000; Williams et al., 2016, 2020). It has therefore been suggested that such instability of sensory representations during seizures may contribute to impairing the cortical functions necessary for normal conscious experience (Williams et al., 2020). Other types of epilepsy are associated with abnormal cortical synchronisation in mammals (Uhlhaas and Singer, 2006). For example, short periods of high-frequency oscillations (100–500 Hz) are frequently observed near the time of seizure onset in rodent models and patients with mesial temporal lobe or neocortical focal epilepsy, where they may signal proximity to the epileptogenic focus (Bragin et al., 1999; Jirsch et al., 2006).

#### 6.2.2 Anoxia

Disturbed cortical dynamics are also characteristic of cerebral ischaemia or anoxia following cardiac arrest, asphyxia or severe stroke. As a metabolically expensive organ, the mammalian brain is very sensitive to any interruption in its oxygenation, and prolonged global anoxia is often associated with a poor prognosis. Loss of oxygenation results in a rapid decrease in the amplitude and frequency of cortical activity, which gives way to a flat or isoelectric EEG. However, convergent findings from clinical investigations and studies on various mammals indicate that this sustained electrocerebral inactivity, marking the entry into a deep comatose state, is generally preceded by stereotyped changes in the frequency content of EEG signals (reviewed in Charpier, 2023).

**Figure 7:**
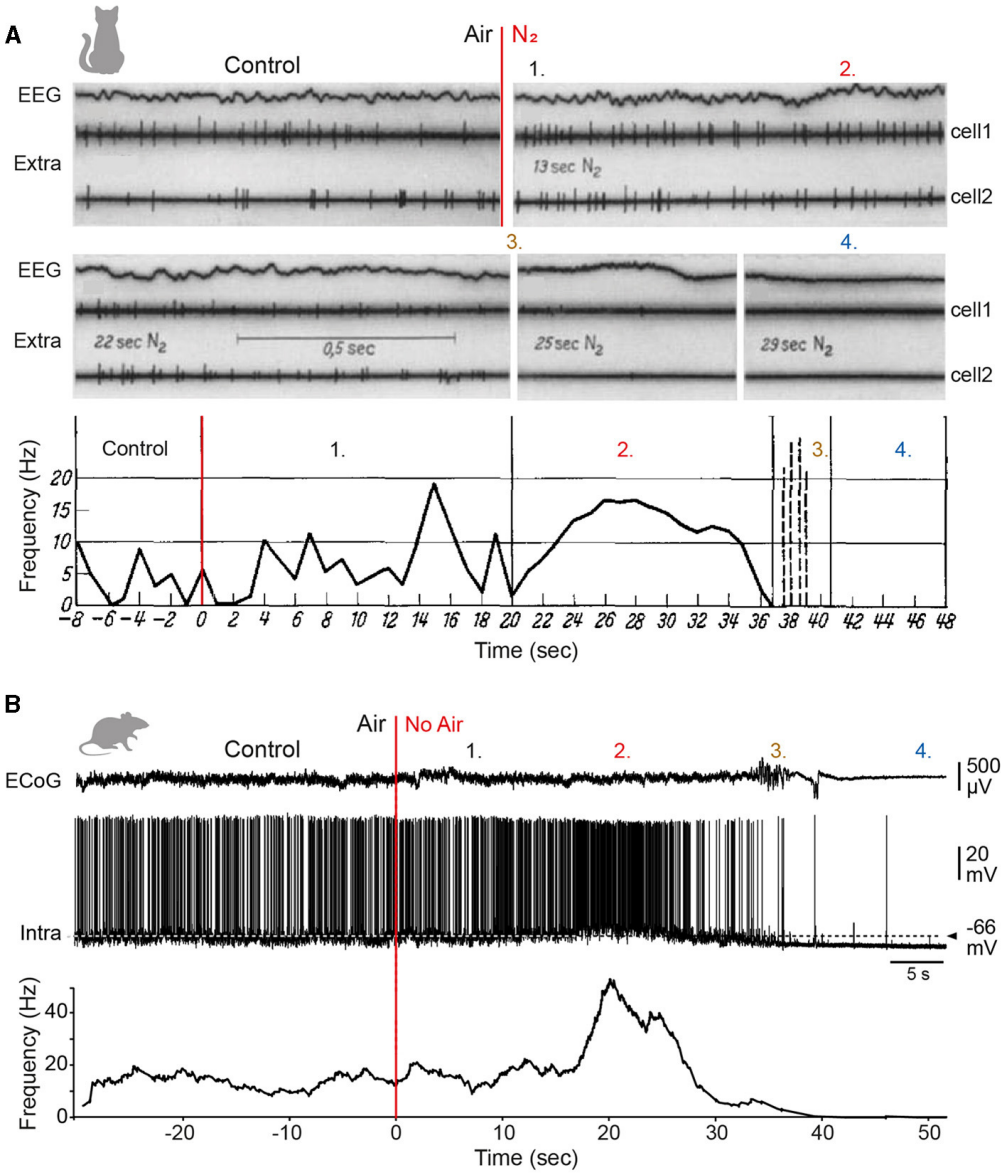
Conservation of anoxia-induced cortical rhythms in mammals. **(A)** EEG and firing activity of two extracellularly recorded neurons (Extra) from the cat visual cortex before (Control) and after induction of global cerebral anoxia by N_2_ inhalation (red line). The first period of relative stability (1.) is followed by a transient cortical activation evidenced by an acceleration of the EEG waves and an increase in the neuronal firing frequency (2.). The EEG activity then slows down and becomes dominated by delta-frequency waves that correlate with weak, irregular firing in cortical neurons (3.). The onset of the isoelectric state marks the loss of cellular and network activity (4.). Modified from Creutzfeldt et al. (1957). **(B)** Simultaneous recordings of the electrocorticogram (ECoG) and intracellular activity of a somatosensory cortex L5 pyramidal neuron (Intra), before and after cessation of artificial ventilation in a sedated and curarized rat. The dashed horizontal line indicates the mean control membrane potential (-66 mV). The graph shows the firing rate as a function of time. The four periods described above in the cat are similarly observed in the rat: the period of stability (1.), the period of firing rate increase (2.), the subsequent period of slow cortical oscillations (3.), and the isoelectric state (4.). Adapted with permission from Carton-Leclercq et al. (2023).

Experiments conducted on sedated or anaesthetised rodents, cats, and monkeys (rat: Hansen, 1978; Borjigin et al., 2013; Schramm et al., 2020; Carton-Leclercq et al., 2023; cat: Sugar and Gerard, 1938; Creutzfeldt et al., 1957; Hossmann and Sato, 1971; monkey: Myers and Yamaguchi, 1977) have shown that the onset of anoxia is associated with a transient increase in beta-gamma activities, often mixed with theta oscillations (Figure 7). Single-unit and intracellular recordings revealed that this period of high-frequency EEG activity correlates with rapid, low-amplitude synaptic fluctuations in deep-layer pyramidal neurons, a slight depolarization of their resting membrane potential and an increase in their firing rate (Figure 7) (Creutzfeldt et al., 1957; Carton-Leclercq et al., 2023). This cortical state is thought to result from an overactivation of synaptic receptors due to an excessive release of glutamate in the interstitial medium (Katchman and Hershkowitz, 1993; Fleidervish et al., 2001), although a complete understanding of the underlying cellular and network mechanisms will require further exploration. Fast electrical activity is also observed on the EEG of a number of critically ill patients after activation of the Do Not Resuscitate-Comfort Care protocol and discontinuation of life-sustaining treatment (Auyong et al., 2010; Norton et al., 2017; Xu et al., 2023). These brief occurrences of arousal-like activity, suggesting the emergence of consciousness without any outward signs of it, have been proposed as the origin of the vivid sensations and paradoxical lucidity associated with near-death experiences (Chawla et al., 2017; Parnia et al., 2023; Xu et al., 2023). However, high-frequency oscillations are a widespread rhythm not specific to conscious experiences, and the rather short duration of this episode of cortical activation has been deemed difficult to reconcile with the prolonged perceptions reported by some experiencers. The persistence of these rapid oscillations up to the moment of the isoelectric state and their resurgence at irregular intervals after its onset are also controversial. They have not been documented in all studies (see Chawla et al., 2017; Vincente et al., 2022; Xu et al., 2023 for report of such late surges of fast activity and Clute and Levy, 1990; Norton et al., 2017; Matory et al., 2021 for their non-occurrence) and it has been suggested that they may, at least in part, reflect muscle contractions rather than brain activity (Greyson et al., 2022).

The high-frequency cortical state is rapidly followed by a gradual decline of cortical activities in all frequency bands, briefly interrupted by a late surge of delta waves. This low-frequency EEG pattern is commonly observed in mammals exposed to anoxia and was first reported in humans by Berger during his pioneering experiments (human: Berger, 1934; Clute and Levy, 1990; Ammirati et al., 1998; Norton et al., 2017; rat: Borjigin et al., 2013; Lee et al., 2017; Schramm et al., 2020; Carton-Leclercq et al., 2023; cat: Sugar and Gerard, 1938; Creutzfeldt et al., 1957; rabbit: Colin et al., 1981). At this stage of the anoxic process, cortical pyramidal neurons show a reduced spontaneous firing and irregular synaptic depolarizations that progressively attenuate in amplitude and frequency with time. This dampening of cellular activity precedes the entry into the isoelectric state, marked by a cessation of spontaneous firing, and a loss of EEG and neuronal voltage fluctuations (Figure 7) (Creutzfeldt et al., 1957; Fujimura et al., 1997; Schramm et al., 2020; Carton-Leclercq et al., 2023). When ATP reserves reach a critical threshold and metabolic impairment is near-complete (85–90% decrease of the normal level), a large amplitude EEG wave, referred to as the “wave of death”, suddenly emerges on the flat EEG. This wave reflects a massive depolarization (anoxic depolarization) of cortical neurons following the inhibition of Na^+^/K^+^ ATPase pumps and the loss of homeostatic control of transmembrane ionic concentrations (Pietrobon and Moskowitz, 2014). Except for its onset time that may differ among species (but direct comparison is difficult due to variations in experimental conditions; Charpier, 2023 for review), anoxic depolarization has been observed with many phenomenological and mechanistical similarities in most mammals (mouse: Takano et al., 2007; rat: van Rijn et al., 2011; Schramm et al., 2020; Carton-Leclercq et al., 2023; human: Carlson et al., 2018; Dreier et al., 2018; rabbit: Leão, 1947; cat: Hossmann, 1971; monkey: Harris et al., 1981), as well as in the central nervous system of several invertebrates (Spong et al., 2016). This suggests that the neural correlates of near-death processes have also been well preserved throughout evolution. It is important to note that the wave of death is no longer considered terminal. Evidence from multi-scale electrophysiological recordings in rodents has shown that it can be reversed by a timely reoxygenation and replaced by its mirror wave, the “wave of resuscitation”, which reflects the progressive repolarization of cortical neurons (Schramm et al., 2020; Carton-Leclercq et al., 2023). Although the wave of resuscitation has not yet been identified in humans, there is good reason to believe that the use of suitable electrodes and broadband recordings that preserve low frequencies will soon enable its discovery.

## 7 Discussion

The data reviewed above highlight the many anatomical disparities that distinguish the neocortex of the different mammals. Evolution has led to variations in the overall size of the brain, the number of cortical areas, the number of neurons, the size of their dendritic trees and the estimated number of synapses, and some of these differences appear to be particularly pronounced in humans. Does this mean that we can talk about unique properties that would set us apart from other species? Probably not. Firstly, because most of these changes are not exclusive to our species but are shared by most large-brained mammals. Secondly, because evidence suggests that the mammalian neocortex is organised into basic computational circuits, with common neuronal types, distribution profiles and synaptic connectivity patterns. This conservation extends to the electrophysiological properties of pyramidal neurons, whose biophysical characteristics (apart from a few specificities such as the expression of the h-current in the supragranular layers), excitability rules and firing profiles are similar across species. Furthermore, the consistency of unitary excitatory synaptic potentials suggests that, despite variations in neuronal architecture, the fundamental transfer of synaptic information between neurons remains unchanged, probably as a result of compensatory processes. Additionally, large-scale brain dynamics, which underpin integrative functions and dysfunctions of the brain, also appear to be largely preserved, pointing to a degree of determinism in the structure-function relationship of neocortical networks. However, variations in the morphology and proportion of certain neuron subtypes between species, individuals or cortical regions argue against the existence of a single, stereotyped mode of operation of the mammalian cortical circuit. To borrow a concept dear to the field of comparative anatomy, it would be more appropriate to refer to a “unity of plan” or “archetypal scheme” from which, on a continuum of possibilities, a number of specialisations emerge to meet different functional requirements. Thus, compared with other species, there is no revolution in the properties of human neurons and neocortical circuits; there are certainly differences, but there is also a marvellous resemblance, reflecting the continuation of an evolution that began with the first mammals and will probably continue beyond our species. In this respect, we could transpose to the study of the organisation and functioning of the neocortex what Darwin (1871) wrote in *The Descent of Man, and selection in relation to sex*: “Whether primaeval man, when he possessed very few arts of the rudest kind, and when his power of language was extremely imperfect, would have deserved to be called man, must depend on the definition which we employ. In a series of forms graduating insensibly from some ape-like creature to man as he now exists, it would be impossible to fix on any definite point when the term ‘man' ought to be used.” Echoing Darwin's thoughts on language evolution, it is interesting to note that left hemispheric asymmetries in regions homologous to human language areas have been observed in non-human primates (Gannon et al., 1998; Cantalupo and Hopkins, 2001). Moreover, research on baboons and chimpanzees has revealed that the direction and degree of lateralization of gestural communication are linked to contralateral asymmetry of part of the cortical region homologous to Broca's area (Taglialatela et al., 2006; Meguerditchian et al., 2012; Becker et al., 2022). These findings suggest a possible evolutionary continuity between primate gestural communication lateralization and human language hemispheric specialisation, implying that the cerebral organisation of communication areas could have been inherited from a common ancestor, before the emergence of speech (Becker et al., 2022). Furthermore, the recent discovery of a complex structure in sperm whale vocalisations (Sharma et al., 2024), as well as the detection of a lateralized arcuate fasciculus (a white matter fibre tract involved in human language) in the bottlenose dolphin (Wright et al., 2018), raise intriguing questions about the existence and function of such hemispheric asymmetry in species that have long diverged from primates.

The analysis of the basic components of the circuit could, however, be considered insufficient to account for the complexity of functioning of the neocortex as a whole. Indeed, the emergentist approach to complex systems postulates that at each level of complexity, entirely new properties—not present in the system's constituents—appear, governed by new laws, new concepts and new generalisations; a theory that could be summed up by the physicist Anderson's eloquent formula “*more is different”* (Anderson, 1972). A well-known example of this theory is that of the properties of water, such as vorticity or entropy, which cannot be deduced from knowledge of the characteristics of individual H_2_O molecules. Applied to the brain, the concept of emergence implies a dynamic and highly integrated functioning of the cerebral cortex, in which the coordinated activity of groups of neurons distributed in primary and associative cortical areas allows the emergence of coherent behavioural and cognitive acts. Direct (though only correlative) evidence for the formation, mediated by synchronisation mechanisms over multiple frequency bands, of such coherent ensembles has been obtained in humans, cats, monkeys, and rats, during a variety of active perceptuomotor behaviours (for a review, see Varela et al., 2001). It seems reasonable to assume that the iteration of an archetypal cortical circuit (considered here as a cognitive module or unit) could increase the number of possible combinations of the different sub-circuits and favour the behavioural diversity of the system. We also need to consider the evolutionary aspect of these complex brain figures, which are likely to change as a function of the physical, biological and social contexts of each species or individual. These dynamic and reciprocal links between the cortex and its environment will thus lead to the emergence of diverse and characteristic experiences, underpinned by common elementary cerebral processes from which they are indissociable.

A balanced synthesis between reductionist and emergentist approaches could postulate that the cerebral activities underlying mental processes are shared by different species and that cognitive performance is the result of complex cerebral dynamics. However, this perspective does not answer the fundamental question of how specific spatio-temporal brain dynamics translate into conscious experiences, such as sensations, reasoning or memories. Like the physiologist du Bois-Reymond (1882) (the first to describe AP in nerve fibres), who identified this issue as one of the seven world enigmas in his famous 1882 lecture on *Ignorabimus*, modern neurophilosophers continue to assert that bridging the explanatory gap between brain activity and subjective phenomena, regardless of the species, will remain beyond the reach of scientific investigation (Levine, 1983; Chalmers, 1995). A more optimistic view would be to consider that this intricate relationship between neural and mental events, which defies the intrinsic limits of our knowledge but whose analysis is a real driving force for questioning, is a missing link whose contours and principles have yet to be defined.

## Author contributions

SM: Conceptualization, Writing – original draft, Writing – review & editing.
